# Katanin p80, NuMA and cytoplasmic dynein cooperate to control microtubule dynamics

**DOI:** 10.1038/srep39902

**Published:** 2017-01-12

**Authors:** Mingyue Jin, Oz Pomp, Tomoyasu Shinoda, Shiori Toba, Takayuki Torisawa, Ken’ya Furuta, Kazuhiro Oiwa, Takuo Yasunaga, Daiju Kitagawa, Shigeru Matsumura, Takaki Miyata, Thong Teck Tan, Bruno Reversade, Shinji Hirotsune

**Affiliations:** 1 Department of Genetic Disease Research, Osaka City University, Graduate School of Medicine, Asahi-machi 1-4-3, Abeno, Osaka 545-8585, Japan; 2 Institute of Medical Biology, Human Genetics and Embryology Laboratory, Singapore; 3 Department of Anatomy and Cell Biology, Nagoya University Graduate School of Medicine, 65 Tsurumai, Showa, Nagoya 466-8550, Japan; 4 Advanced ICT Research Institute, National Institute of Information and Communications Technology, Kobe, Hyogo 651-2492, Japan; 5 CREST, Japan Science and Technology Agency, Chiyoda-ku, Tokyo 102-0076, Japan; 6 Graduate School of Life Science, University of Hyogo, Harima Science Park City, Hyogo 678-1297, Japan; 7 Department of Bioscience and Bioinformatics, Faculty of Computer Science and Systems Engineering, Kyushu Institute of Technology, Kawazu 680-4, Iizuka, Fukuoka 820-850, Japan; 8 JST-SENTAN, 4-1-8, Honcho, Kawaguchi, Saitama 332-0012, Japan; 9 JST-CREST, 4-1-8, Honcho, Kawaguchi, Saitama 332-0012, Japan; 10 Division of Centrosome Biology, Department of Molecular Genetics, National Institute of Genetics, 1111 Yata, Mishima, Shizuoka, 411-8540, Japan; 11 Department of Cell Biology, Institute for Virus Research, Kyoto University, Sakyo-ku, Kyoto, 606-8507, Japan; 12 Institute of Molecular and Cellular Biology, A*STAR, Singapore 138648, Singapore; 13 Department of Paediatrics, National University of Singapore, Singapore 119260, Singapore

## Abstract

Human mutations in *KATNB1* (p80) cause severe congenital cortical malformations, which encompass the clinical features of both microcephaly and lissencephaly. Although p80 plays critical roles during brain development, the underlying mechanisms remain predominately unknown. Here, we demonstrate that p80 regulates microtubule (MT) remodeling in combination with NuMA (nuclear mitotic apparatus protein) and cytoplasmic dynein. We show that p80 shuttles between the nucleus and spindle pole in synchrony with the cell cycle. Interestingly, this striking feature is shared with NuMA. Importantly, p80 is essential for aster formation and maintenance *in vitro*. siRNA-mediated depletion of p80 and/or NuMA induced abnormal mitotic phenotypes in cultured mouse embryonic fibroblasts and aberrant neurogenesis and neuronal migration in the mouse embryonic brain. Importantly, these results were confirmed in p80-mutant harboring patient-derived induced pluripotent stem cells and brain organoids. Taken together, our findings provide valuable insights into the pathogenesis of severe microlissencephaly, in which p80 and NuMA delineate a common pathway for neurogenesis and neuronal migration via MT organization at the centrosome/spindle pole.

A prominent trait of the neocortex is its complex yet elaborately organized cellular architecture, the formation of which relies on strictly regulated neurogenesis and neuronal migration. The proliferation of neural progenitor cells (NPCs) during the early stages of neocortex formation is critical to expand the progenitor pool at the ventricular zone, and subsequent mitotic divisions result in the generation of post-mitotic neurons, which then migrate to the cortical plate in an “inside-out” manner^1^. Numerous genes are involved in the regulation of the onset, progression, and termination of corticogenesis. Abnormalities in these processes lead to human diseases, which are often characterized by different forms of mental retardation or cognitive disabilities and severe epilepsy^2^. Therefore, investigations of cortical malformations substantially contribute to the understanding of normal brain development and disease.

Microcephaly and lissencephaly have traditionally been classified as distinct forms of congenital neurodevelopmental disorders, with each disorder encompassing a genetically heterogeneous group of monogenic disorders. Autosomal-recessive primary microcephaly (MCPH) manifests as an architecturally normal brain that is reduced in size. Multiple genes mutated in MCPH encode proteins that localize to the centrosome or mitotic spindle poles^3^,^4^. Many of these genes have also been implicated in the regulation of progenitor cell-cycle progression and the determination of whether progenitors will continue to proliferate or differentiate into post-mitotic neurons^3^,^4^. In contrast to microcephaly, lissencephaly is caused by a reduced number or absence of gyri and sulci of the cortical surface within a normal brain volume. This phenotype reflects an abnormal histological organization of the cortical layers as a result of neuronal migration disruption^2^,^5^. The identified genetic causes of lissencephaly include mutations in *LIS1*^6^,^7^, *DCX*^8^, *RELN*^9^ and *TUBA1A*^10^. Microlissencephaly (i.e. microcephaly with various architectural defects of the cortex) has been associated with mutations in *WDR62*^11^,^12^ and *NDE1*^13^. Recently, mutations in *KATNB1* (p80), which encodes the non-catalytic regulatory p80 subunit of katanin^14^,^15^,^16^,^17^, have been shown to cause severe microlissencephaly^18^,^19^. These findings highlight the critical functions of *KATNB1, WDR62* and *NDE1* during neurogenesis and neuronal migration which suggest the existence of a common pathophysiological pathway responsible for microcephaly and lissencephaly.

Katanin, a heterodimer of p60 and p80, is a microtubule (MT)-severing enzyme^14^. The p60 subunit exhibits ATP-dependent enzymatic activity, whereas p80 is reported to target p60 to the centrosome^17^. Recent studies have documented a novel regulatory function for p80 during cortical cerebral development in different animal models, including mice and zebrafish. In particular, p80 has been determined to regulate the overall number of centrioles and cilia and is necessary for Hedgehog signaling during neocortical development.

In this study, we demonstrate that p80 is essential for the proper regulation of MT dynamics at the centrosome/spindle pole in combination with cytoplasmic dynein and NuMA (nuclear mitotic apparatus protein). Cytoplasmic dynein is a MT-associated molecular motor that moves in a minus-end-directed fashion^20^. The intracellular functions of dynein include vesicular and organelle transport, positioning of intracellular organelles, and various aspects of mitotic spindle dynamics^20^. NuMA is a component of the polar region of the mitotic apparatus^21^. NuMA is essential for tethering spindle MTs to their poles, and for spindle positioning in asymmetric cell division^22^. We identify NuMA as a p80-interacting partner and document that both proteins shuttle between the nucleus and spindle pole in synchrony during the cell cycle. *In vitro* studies using patient-derived induced pluripotent stem cells that carried *KATNB1* mutations and siRNA-mediated knockdowns indicated a novel function for p80 in centrosome/spindle pole formation and maintenance. In a cell-free reconstitution assay, the combination of p80, NuMA and cytoplasmic dynein, was sufficient to trigger aster formation and maintenance. This result was corroborated *in vivo* by decreased neurogenesis and neuronal migration in mouse embryonic brains. Together, our findings indicate a common pathogenesis for microcephaly and lissencephaly driven by dysregulated MT dynamics at the centrosome/spindle pole.

## Results

### p80 interacts with NuMA and regulates cytoplasmic dynein

**Figure 1:**
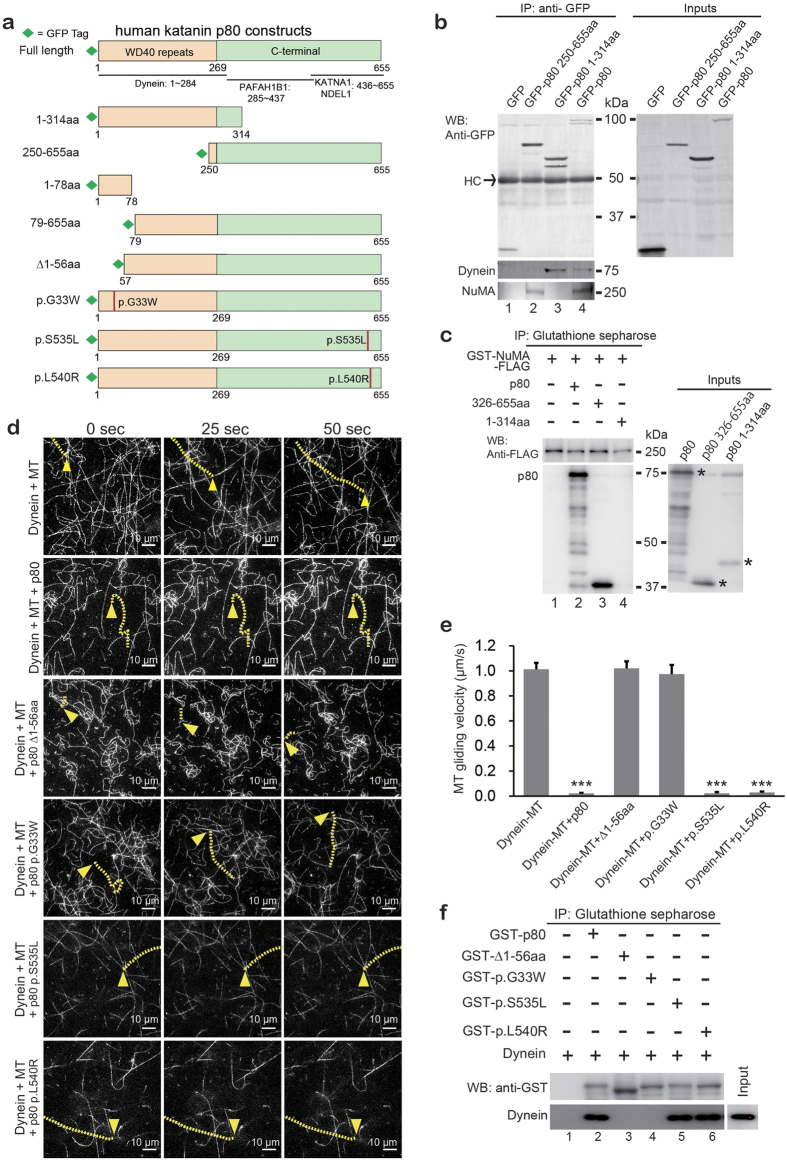
Interaction of p80 with NuMA and cytoplasmic dynein. (**a**) Schematic diagram of p80 subunit of katanin with previously mapped interaction sites. All GFP-fusion fragments including four patient-derived mutation forms^18^,^19^ (lower four fragments) used in this study are presented. (**b**) Immunoprecipitation assay with MEF cell lysates that expressed GFP or GFP-fusion p80 constructs. Proteins co-precipitated by anti-GFP antibody were examined via WB. As indicated, GFP or GFP-fusion p80 fragments (upper panel) co-precipitated dynein (middle panel, lanes 3 and 4) and NuMA (lower panel, lanes 2 and 4). Inputs indicate 10% of each cell lysate used for this immunoprecipitation assay. (**c**) GST pull-down assay with 0.2 μM GST-NuMA-FLAG. NuMA prebound to glutathione Sepharose was incubated with 0.5 μM full-length p80, p80 326-655 aa or p80 1-314 aa. Inputs indicate 10% of each recombinant protein used for this GST pull-down assay. Asters in input membrane indicate each recombinant protein band. (**d**) Dynein motility affected by p80 or its mutant forms derived from patients. Dynein-driven MT gliding (upper panel and Supplementary Video 1) was arrested by the addition of p80 (second panel and Supplementary Video 2), p80 p.S535 L or p80 p.L540R (lower two panels and Supplementary Videos 5–6), but not by the addition of p80 ∆1-56 aa or p80 p.G33W (middle two panels and Supplementary Videos 3–4). MT gliding velocity was quantified and is presented in (**e**). One example of MT tracking in each panel is colored in yellow. Arrowheads indicate the start points of the marked MTs. Scale bar, 10 μm. (**e**) Quantified dynein-driven MT gliding. Fifty MTs from 5 independent movie files were analyzed for each experiment; *P*-values were calculated using analysis of variance (ANOVA), mean ± s.e., ***P < 0.001. There were no statistically significant differences between two groups: an unaffected group that contained N-terminal mutated ∆1-56 aa and p.G33W and a remarkably affected group that contained p80, p.S535 L and p.L540R. (**f**) GST pull-down assay with 0.3 μM full-length p80 or p80 mutations. Target proteins prebound to glutathione Sepharose were incubated with 0.5 μM cytoplasmic dynein. Input lane indicates 5% of dynein used for this GST pull-down assay.

To identify the partners that interact with p80, we performed direct co-immunoprecipitation (Co-IP) of mouse brain lysates, followed by mass spectrometric analysis. NuMA was identified as a p80 binding protein, along with cytoplasmic dynein (Supplementary Fig. S1a and Table S1). The binding of cytoplasmic dynein by the N-terminal WD40 repeat domain of p80 has previously been reported by our group^23^. A previous proteomic analysis had suggested the interaction between NuMA and p80^24^; however, their direct binding evidence had not been reported. To confirm these findings, GFP or GFP-conjugated p80 fragments (Fig. 1a) were overexpressed in mouse embryonic fibroblast (MEF) cells, and Co-IP was performed using an anti-GFP antibody (Fig. 1b, upper panel). Both cytoplasmic dynein (middle panel, lanes 3,4) and NuMA (lower panel, lanes 2 and 4) were pulled down by full-length p80. The N-terminal WD40 repeat domain (1–314 aa) of p80 preferentially bound to cytoplasmic dynein, whereas its C-terminal region (250–655 aa) preferentially bound to NuMA (Fig. 1b). To investigate the direct interaction of p80 and NuMA, we performed an *in vitro* pull-down assay using recombinant proteins of p80 and NuMA and demonstrated that p80 directly interacts with NuMA via its C-terminus without a requirement for dynein (Fig. 1c).

Another established binding partner of p80 is LIS1^23^. Similar to the case of p80, mutations in the WD40 repeat domain in LIS1 cause lissencephaly^6^,^7^. We have previously reported that LIS1 suppresses the motility of cytoplasmic dynein on MTs, which is essential for the anterograde transport of cytoplasmic dynein^25^. This LIS1 activity may also be involved in processes that are essential for proper migration in neurons (e.g., the stabilization of MT bridging between the centrosome and nucleus and the capture of MTs at the nuclear envelope)^26^. Therefore, we investigated whether p80 also suppresses the motility of cytoplasmic dynein using an MT gliding assay. Remarkably, similar to LIS1, p80 arrested the MT gliding activity of cytoplasmic dynein (Fig. 1d, upper two panels, Fig. 1e and Supplementary Videos 1,2). To determine whether this activity may have pathological relevance, we investigated whether p80 with the mutations identified in microlissencephalic patients retains the ability to suppress the motility of cytoplasmic dynein. We initially tested the interaction and regulatory activity of pathogenic alleles that code for an N-terminally truncated p80 protein (c.1A > G, Δ1–56 aa) or an N-terminal missense (c.97G > T, p.G33W)^18^. Strikingly, both mutant forms of p80 had a reduced affinity to dynein (Fig. 1f, lanes 2–4) and no longer inhibited its activity (Fig. 1d, middle two panels, Fig. 1e and Supplementary Videos 3–4). In contrast, disease-causing missense mutations located at the C-terminal of p80, far from the dynein binding region (c.1606T > G, p.S535L and c.1619G > T, p.L540R)^19^, continued to interact with dynein *in vitro* (Fig. 1f, lanes 5,6), and retained the ability to suppress MT gliding of dynein (Fig. 1d, lower two panels, Fig. 1e and Supplementary Videos 5–6). These novel interactions and functions of p80 with NuMA and dynein may shed important insights regarding the involvement of p80 in brain development and may also provide an explanation for the phenotypic variability that occurs among *KATNB1* mutant patients^18^,^19^.

### p80 alternates localization between centrosome/spindle pole and nucleus

**Figure 2:**
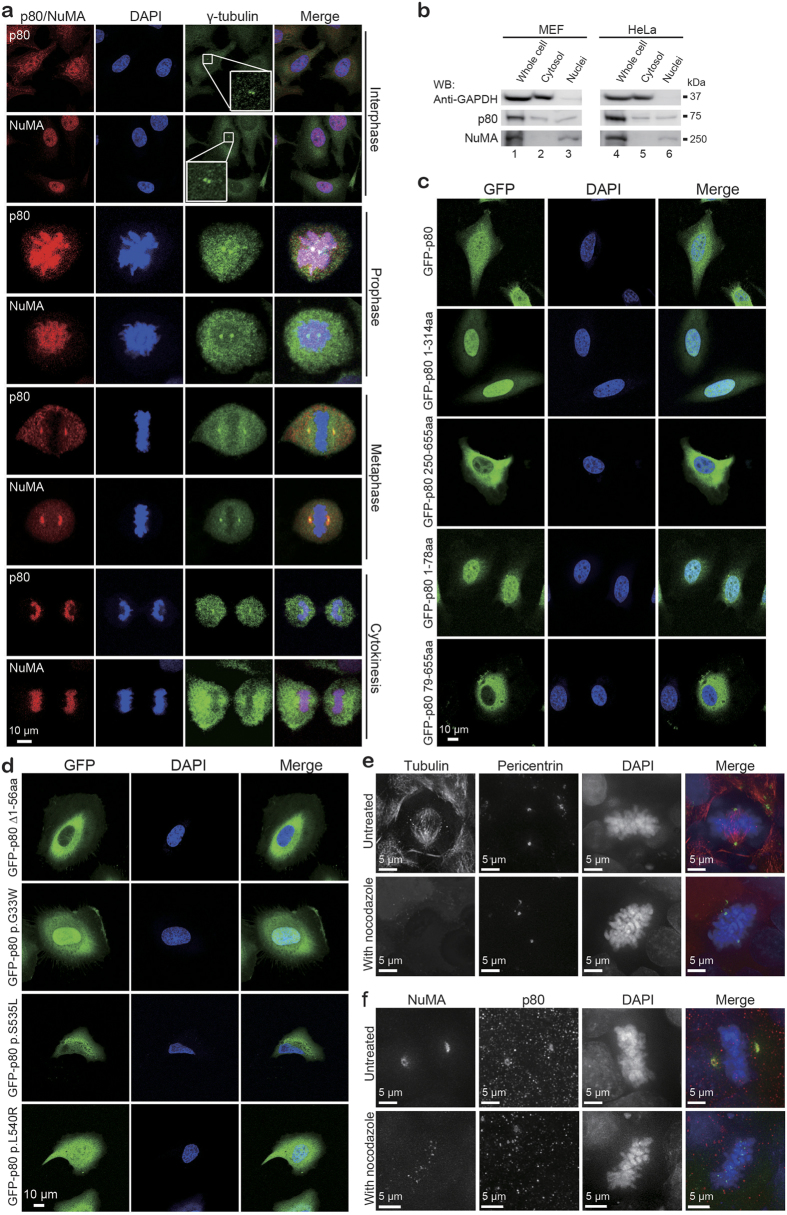
Subcellular localization of p80 in HeLa cells. (**a**) Intracellular localization of endogenous p80 and NuMA at different mitotic stages. Synchronized and thymidine-released HeLa cells, as indicated, were probed with antibodies against p80^23^ or NuMA with γ-tubulin. Cell nuclei were visualized with 4′,6-diamidino-2-phenylindole (DAPI) and γ-tubulin probed centrosomes and spindle poles. Insets are from rectangle-surrounded area that indicates centrosomes in the interphase cells. (**b**) Expression of p80 and NuMA in MEF and HeLa cells. Approximately 5 × 10^6^ MEF or HeLa cells were collected and homogenized with NE-PER nuclear and cytoplasmic extraction reagent (Thermo Fisher Scientific, MA, USA) according to the manufacturer’s instructions. Cytosolic and nuclear extracts were adjusted to a 2 mg/mL concentration and were subsequently analyzed via WB with antibodies against glyceraldehyde-3-phosphate dehydrogenase (GAPDH, upper), p80 (middle) or NuMA (lower). As indicated, GAPDH and NuMA were expressed in cytosolic and nuclear extractions, respectively, whereas p80 was identified in both cytoplasmic and nuclear extracts. GAPDH was used as a cytosolic marker. (**c**) Expression of GFP-p80 constructs in HeLa cells. GFP-p80 constructs (Fig. 1a) were transfected into HeLa cells, and each subcellular localization was examined. (**d**) Expression of four mutant forms of p80 in HeLa cells obtained from patients. GFP-p80 Δ1-56 aa (uppermost panel) exhibited only cytoplasmic expression, whereas GFP-p80 p.G33W, GFP-p80 p.S535 L and GFP-p80 p.L540R exhibited the same distribution pattern as full-length p80. Scale bar, 10 μm. (**e,f**) p80 and NuMA co-localization in metaphase spindle pole. Metaphase iPSCs were treated with 300 ng/ml nocodazole for 1 h at 4 °C, and pericentrin (**e**), p80 and NuMA (**f**) localizations were probed using antibodies against tubulin, pericentrin, p80 and NuMA. Nuclei were probed with DAPI. Scale bar, 5 μm.

Katanin is an MT-stimulated ATPase and requires ATP hydrolysis to sever MTs at the centrosome^14^,^17^. Previous findings have indicated that the WD40 repeats of p80 katanin are sufficient to target GFP-fusion proteins to the centrosome^17^. We have also reported that similar to LIS1 and NDEL, p80 is dynamically redistributed during neurogenesis and neuronal migration^23^. These proteins are restricted to the centrosome during proliferation; however, they redistribute to the leading processes in migrating neurons^23^. Thus, p80 may have roles in addition to its centrosomal activities. Here, we identified NuMA as a protein that interacts with p80 (Fig. 1b,c and Supplementary Fig. S1a). NuMA is an important structural component in both the spindle pole and nucleus, depending on the cell cycle phase^22^. Therefore, we revisited the subcellular distribution of endogenous p80 throughout the cell cycle in HeLa cells. To our surprise, endogenous p80 dynamically changed its localization during the cell cycle (Fig. 2a). In interphase, p80 was present in both the cytosolic and nuclear compartments (uppermost panel and Supplementary Fig. S1b), whereas in early mitosis, p80 was predominantly located within the nucleus. During metaphase, p80 moved to the spindle pole, following which it was redistributed to the sister chromatids during cytokinesis. After mitosis, p80 traveled back into the nucleus and cytoplasm (Fig. 2a and Supplementary Fig. S1b). To the best of our knowledge, this study represents the first report of p80 as a transient nuclear protein. Intriguingly, the redistribution pattern of NuMA exhibited similar characteristics to p80 throughout the cell cycle (Fig. 2a). The nuclear co-localization of p80 and NuMA was also confirmed by blotting protein extracts from MEF and HeLa cells (Fig. 2b, lanes 3 and 6) as documented via immunocytochemistry (Fig. 2a). We further investigated the p80 and NuMA expression in migrating granular neuronal cells and determined that both proteins are expressed in the perinuclear region and in nuclei (Supplementary Fig. S1c–e). This cellular distribution indicates novel possibilities regarding to the involvement of these proteins in migrating neurons.

We subsequently determined the regulatory region of p80 for nuclear localization using GFP-conjugated p80 constructs (Fig. 1a). Using bioinformatic tools, a clear nuclear localization sequence (NLS) for p80 could not be identified. Similar to endogenous p80 (Fig. 2a), GFP-conjugated human p80 (GFP-p80) was localized in the nucleus and in the cytoplasm of interphase cells (Fig. 2c, uppermost panel). To further investigate the p80 nuclear-targeting region, various GFP-tagged constructs, including the p80 amino acids (aa) 1-314 (N-terminal WD40 repeats), 250–655 (C-terminal), 1–78 (*Sac*I site) and 79–655 (*Sac*I and *Kpn*I sites) were cloned and transfected in HeLa cells (Figs 1a and 2c). The GFP-p80 1–314 aa and GFP-p80 1–78 aa were mainly targeted to and accumulated in the nucleus, whereas the GFP-p80 250–655 aa and GFP-p80 79–655 aa were predominately localized in the cytoplasm (Fig. 2c). We further examined the subcellular distribution of four mutant forms of p80 from patients. Interestingly, the GFP-p80 Δ1-56 aa was excluded from the nucleus, whereas other mutated GFP-p80s exhibited normal nuclear targeting (Fig. 2d). These findings suggest that the N-terminal amino acids 1–56 of p80 are required for its nuclear localization.

Intriguingly, during metaphase, NuMA is localized along the spindle poles as a gradient with the strongest staining around the centrosomes, whereas p80 exhibited diffused staining all over the cytoplasm with strong staining around the centrosomes (Fig. 2a). The co-localization of p80 and NuMA around the centrosome suggests that they may play an important role during mitosis. To determine their localization during metaphase, MT disrupting experiments were performed using nocodazole. When human induced pluripotent stem cells (iPSCs) undergoing metaphase were treated with nocodazole for 1 h, p80 and NuMA were diffusely distributed throughout the cell in contrast to pericentrin (Fig. 2e,f). p80 and NuMA co-localization in the spindle pole was also confirmed with 3D alignment measurements in iPSCs. Both p80 and NuMA were located around the centrosome and exhibited significant co-localization with each other (Pearson’s coefficient in ROI volume = 0.67), but not with pericentrin, which was localized in a deeper pericentriolar material layer (Supplementary Fig. S1f,g). This distribution during early mitosis suggests that similar to NuMA, p80 regulates MT dynamics at the spindle pole and may participate in mitotic spindle assembly.

### p80 and NuMA are essential for normal mitotic spindle assembly

**Figure 3:**
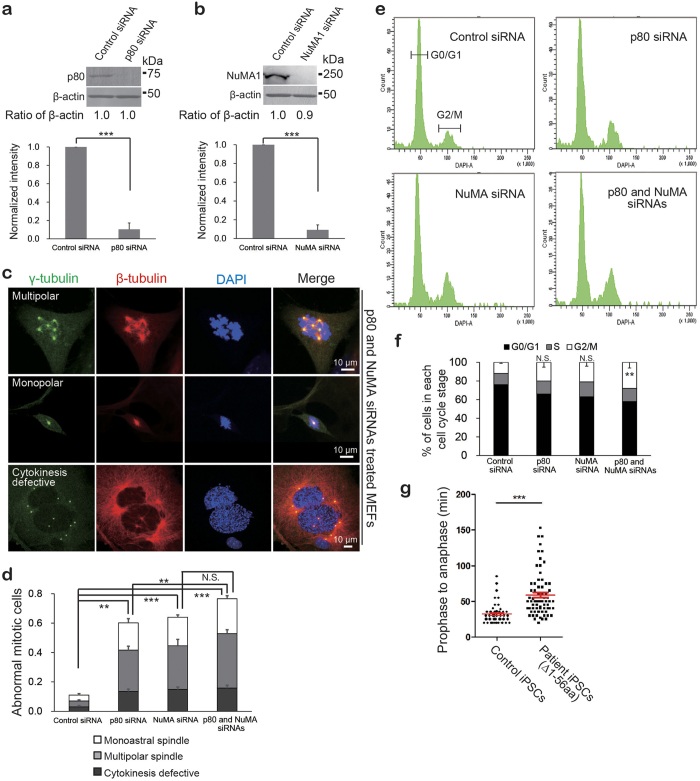
Mitotic-defective phenotypes caused by depletion of p80 or NuMA in MEF cells. (**a,b**) siRNA-mediated knockdown of p80 (**a**) and NuMA (**b**) in MEF cells. All siRNA-transfected MEF cell lysates were analyzed via WB using antibodies against p80, NuMA and β-actin. Lower quantitative graphs (*n* = 3) indicate that siRNA treatment effectively decreased p80 (**a**) or NuMA (**b**) expression in both cases. β-Actin was used as a loading control. (**c,d**) Abnormal mitotic phenotypes in p80 (Supplementary Fig. S2a,b), NuMA (Supplementary Fig. S2c,d) or double depletion cells (**c** and Supplementary Fig. S2e) and their quantitative analysis (**d**). siRNA treated cells were stained with anti-γ tubulin antibody (green) to probe centrosomes/spindle poles and anti-β tubulin antibody (red) to probe MT skeletons. As indicated, cells with the depletion of the target proteins exhibited multipolar (upper panels) and monopolar (middle panels) spindles, accompanied by cytokinesis defects (lower panels). These aberrant mitotic phenotypes were analyzed to corroborate the functions of p80 and NuMA in the cell cycle. The results were obtained from three independent experiments, *n* = 329 for control, *n* = 352 for p80 siRNA, *n* = 310 for NuMA siRNA and *n* = 302 for both siRNA treatments (**d**). (**e,f**) Cell cycle profiles in target siRNA-treated MEF cells. Binucleated cells caused by siRNA were analyzed via FACS (**e**) and quantified (**f**), (*n* = 3). (**g**) Quantified data of prophase-to-anaphase length in control and patient iPSCs (Δ1-56 aa). Scale bar, 10 μm. *P*-values were calculated using Student’s *t*-test or analysis of variance (ANOVA), mean ± s.e., *P < 0.05, **P < 0.01, ***P < 0.001.

Mutations in p80 result in defective centrosomes that cause abnormal spindle formation and further lead to cell cycle perturbation^18^. To determine whether p80 and NuMA function together during spindle formation, we designed effective siRNAs (Fig. 3a,b, and Supplementary Fig. S1h,i), and depleted p80 and/or NuMA in MEFs (Fig. 3c–f and Supplementary Fig. S2). Consistent with a previous report^18^, the knockdown of p80 caused a significant increase in the proportion of cells with abnormal spindles, such as multipolar or monoastral spindles, and cytokinesis-defective cells (Fig. 3d and Supplementary Fig. S2a,b). We identified a similar increase in spindle and cytokinesis defects when NuMA alone was knocked down (Fig. 3d and Supplementary Fig. S2c,d) and when p80 and NuMA were knocked down together (Fig. 3c,d and Supplementary Fig. S2e). These findings suggest that p80 and NuMA are both required for normal spindle assembly, and they function in the same pathway during mitosis.

Centrosomes are crucial for proper spindle assembly, and the loss of p80 function causes aberrant numbers of centrosomes^18^. Consistent with our previous results, the depletion of p80 significantly increased the proportion of interphase cells that contained more than two centrosomes compared with control siRNA-treated cells (Supplementary Fig. S2f,h). This phenomenon was also identified in NuMA-depleted cells (Supplementary Fig. S2f,h). This defect was rescued by the co-expression of human p80 or human NuMA (Supplementary Fig. S2g,h). To understand how these defects in centrosome structure and spindle assembly affect the cell cycle, we performed a cell cycle analysis on p80 and/or NuMA knockdown MEF cells, in which the DNA content was measured using flow cytometry. The knockdown of p80 and NuMA significantly increased the cell population in G2/M phase (Fig. 3e,f), which is indicative of cell cycle perturbation.

To understand the mitotic abnormalities that occur in patient cells, carrying the biallelic c.1A > G, Δ1–56 aa mutations^18^, we performed live cell imaging using Hoechst and MT-specific (SiR-tubulin) dyes. Consistent with our previous findings, approximately 50% of the mutant cells exhibited spindle abnormalities, including monoastral spindles, and mitotic abnormalities, such as prolonged metaphase, cytokinesis failure and an asymmetric distribution of the chromosomes (Supplementary Fig. S3, Fig. 3g and Supplementary Videos 7,8,9,10). Strikingly, mitotic spindles frequently collapsed into monoastral spindles with two centrosomes (Supplementary Fig. S3a,b and Supplementary Video 10), which suggests that similar to NuMA^27^, p80 is important for the maintenance of the spindle apparatus during metaphase. Moreover, cells that completed mitosis exhibited significantly prolonged metaphase (Fig. 3g, Supplementary Fig. S3c and Supplementary Videos 7–8). However, apoptosis was identified during live cell imaging at a higher frequency than expected, which suggests an exacerbation of the phenotype by the cytotoxic properties of DNA and tubulin dyes. Together, these findings suggest that p80 and NuMA play a central role during metaphase, and their absence induces abnormal spindle formation, which may perturb the cell cycle by delaying entry into anaphase via checkpoint-dependent mechanisms.

### Knockdown of p80 and NuMA in mouse embryonic brains lead to aberrant neurogenesis and defective neuronal migration

**Figure 4:**
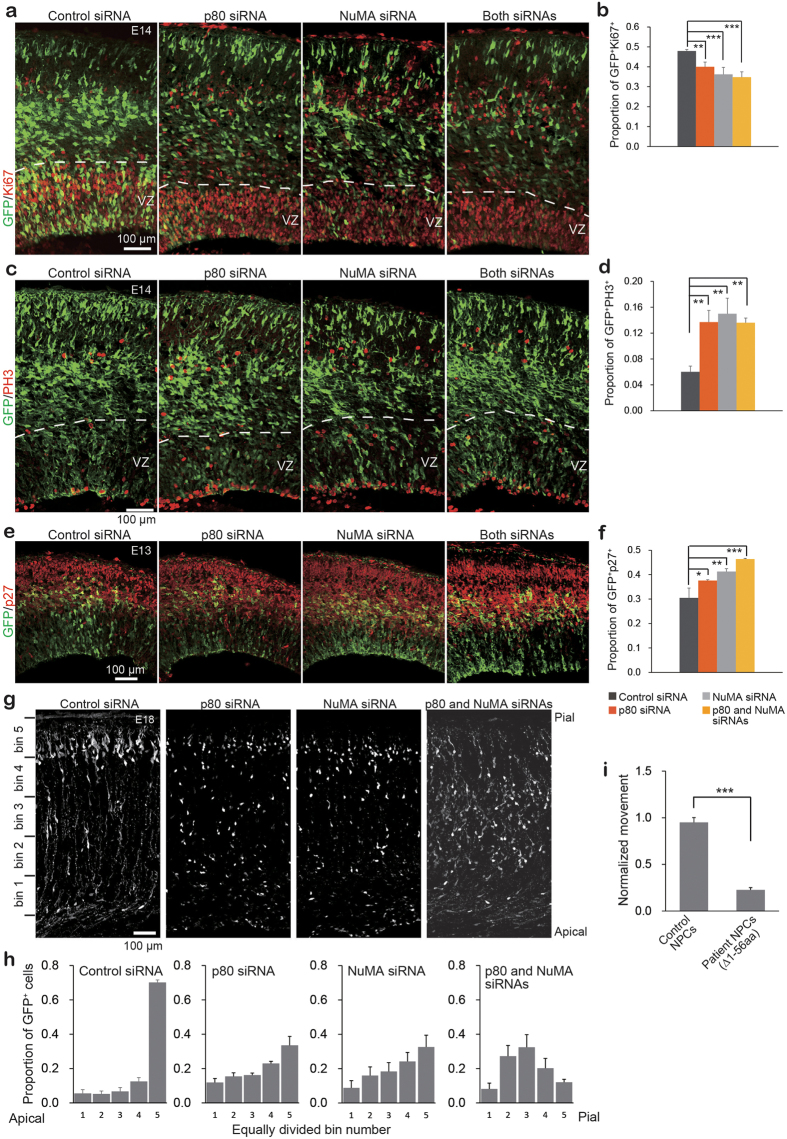
Regulatory function of p80 and NuMA during embryonic brain development. (**a–f**) Reduction in number of proliferating cells attributable to the depletion of p80 or NuMA. The GFP expression vector with control or target siRNA was electroporated at E12, and the proliferative properties were examined at E14 or E13. Coronal brain sections at E14 were stained with anti-Ki67 (**a**) and anti-PH3 (**c**) antibodies to identify actively proliferating cells in the ventricular zone (VZ). At E13, the brains were probed with anti-p27 (**e**) to identify cells that had exited the cell cycle. Quantitative analyses that correspond to Ki67 (% of GFP^+^ Ki67^+^/GFP^+^ in VZ), PH3 (% of GFP^+^ PH3^+^/GFP^+^ in VZ), and p27 (% of GFP^+^ p27^+^/GFP^+^) are available in (**b**), (**d**) and (**f**), respectively. (**g**) Effects of p80 and NuMA on neuronal migration. GFP with control or target siRNA was introduced at E14 and investigated at E18. Coronal sections were stained with anti-GFP antibody (white), and their distributions were analyzed in five equally divided bins as indicated on the left side. Each quantitative analysis was plotted in (**h**). *n* = 3 brains per analysis, scale bar, 100 μm. (**i**) Cell motility assay was performed using an IX83 Live-cell inverted Olympus microscope. For each analysis (*n* = 97 cells), the center of the cell was manually tracked, and the cell movement was calculated using ImageJ manual tracking. Patient-derived NPCs (c. 1A > G, Δ1-56 aa)^18^ were used (Supplementary Videos 13–14), and a quantitative analysis was performed. *P*-values were calculated with ANOVA or Student’s *t*-test, mean ± s.e., *P < 0.05, **P < 0.01, ***P < 0.001.

p80 is highly abundant in the developing mouse cerebral cortex, and human mutations in *KATNB1* induce severe cortical abnormalities^18^,^19^. This evidence indicates that p80 regulates key events during brain development. To further investigate this possibility, we performed *in utero* electroporation of mouse embryonic brains at E12 or E14 to co-transfect neurons with GFP-marker plasmid with p80 and/or NuMA siRNAs. Forty-eight hours post-electroporation, E14 brains were collected and analyzed for actively cycling Ki67-positive progenitor cells in the ventricular zone. Forty-eight percent of the cells in the control brains were Ki67-positive, whereas the loss of p80 or NuMA reduced the percentage of proliferating cells to 40% and 36.2%, respectively, and to 34.8% in the double knockdown brains (Fig. 4a,b). We also documented phospho-histone 3 (PH3), which marks cells undergoing mitosis in the metaphase stage. We identified an increase in PH3-positive cells in the ventricular zone, where neural progenitor cells (NPCs) reside, in p80- and/or NuMA-depleted brains (Fig. 4c,d). This seemingly contradictory result may be explained by the findings that although there are fewer proliferating cells, there may be more cells delayed in the metaphase stage, as identified *in vitro* (Fig. 3g). Furthermore, when we examined brains at E13 using an antibody against p27, a characteristic marker of post-mitotic neurons^28^, we determined that the knockdown of p80 and/or NuMA significantly increased the population of p27-positive cells in the brains (Fig. 4e,f). These findings suggest that in the absence of p80 or NuMA, NPCs are forced to exit the proliferation phase and prematurely commit to neuronal differentiation. Apoptosis also occurred in patient-derived NPCs (c.1A > G, Δ1-56 aa)^18^ as shown via nuclear fragmentation (Supplementary Videos 11–12), which indicates that p80 is important for cell survival.

In addition to MCPH, humans and mice carrying a germline mutation in p80 exhibit structural abnormalities in the brain, which indicate defects in neuronal migration. To determine whether p80 and NuMA are required for neuronal migration, we downregulated their expression in ventricular cells at E14. The migration of transfected neurons towards the pial surface was recorded at E18. The single knockdown of p80 or NuMA led to delayed migration with a substantial number of cells remaining in the deeper apical layers further away from the pial surface. This effect was compounded in the double NuMA and p80 knockdown (Fig. 4g,h). Together, these findings indicate that p80 and NuMA are involved in neuronal progenitor proliferation and survival and are also critical for the proper migration of post-mitotic neurons.

**Figure 5:**
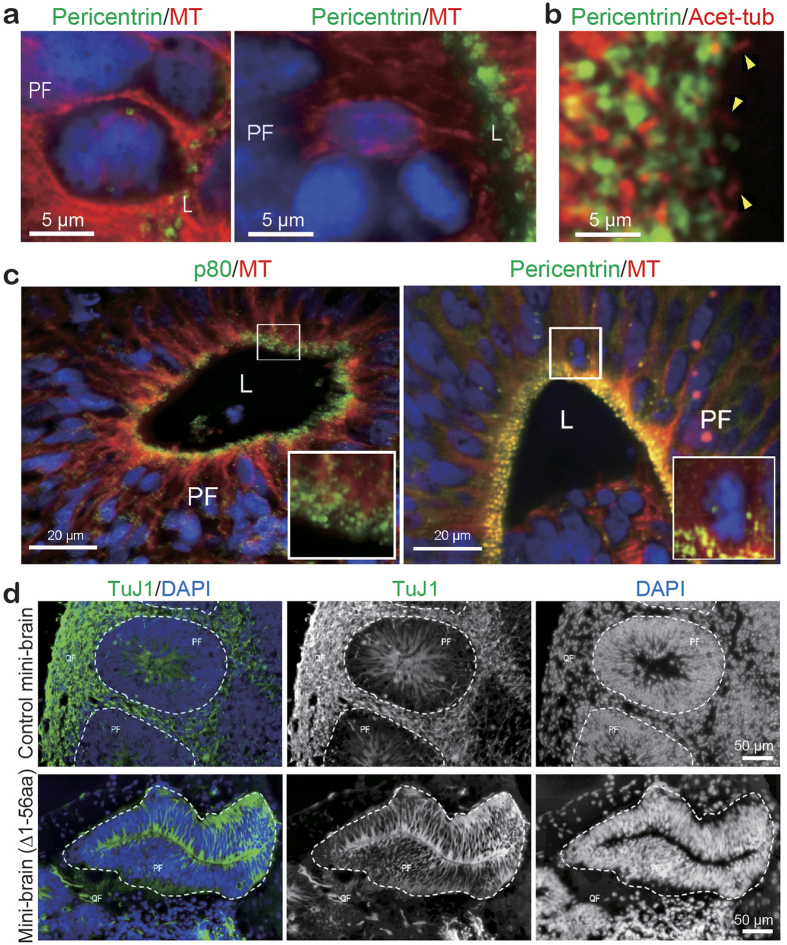
Staining of differentiated mini-brains for the indicated markers. (**a**) Consistent with interkinetic nuclear migration, nuclei in the proliferating field (PF) undergo mitosis near the lumen (left panel), and some nuclei are encaged in MTs when they are away from the lumen (right). Nuclei and MTs were probed with DAPI and the indicated antibodies. Scale bar, 5 μm. (**b**) Cilia (arrowheads) probed with acetylated tubulin are pointing towards the lumen. Scale bar, 5 μm. (**c**) The PF consists of elongated radial-glial-like cells probed with p80/MT (left) and pericentrin/MTs (right). Pericentrin and p80 are arranged around a lumen (L). Rectangle-surrounded areas were enlarged and are presented as insets. Scale bar, 20 μm. (**d**) Post-mitotic neurons (green) in a differentiated patient (c. 1A > G, Δ1-56 aa)^18^ and non-affected control mini-brains. Compared with the non-affected control, the patient’s mini-brain exhibits fewer neurons. PF, proliferating field; QF, quiescent field; L, lumen; MT, microtubule. Scale bar, 50 μm.

Human microcephalic patients with germline mutations in *KATNB1* exhibit milder phenotypes than complete knockout *Katnb1*^*−/−*^ mice or zebrafish, which is consistent with hypomorphic alleles^18^. To understand how these milder hypomorphic mutations affect neuronal migration, we differentiated patient-derived iPSCs into a homogeneous population of Nestin^+^ NPCs (Supplementary Fig. S4a) and performed live cell imaging to track their migration. Compared with the wild-type NPCs (Supplementary Video 13), the patient-derived NPCs (c.1A > G, Δ1-56 aa)^18^ exhibited an impaired cell motility (Supplementary Video 14 and Fig. 4i) in conventional 2D cultures. To more accurately model *in vivo* neuronal migration, we generated 3D cerebral organoids, also referred to as mini-brains^29^ from control and patient-derived iPSCs. After 4–5 weeks of differentiation, the control mini-brains exhibited two distinctly organized structures: TuJ1^*−*^/PCNA^+^ proliferating fields (PF) and TuJ1^+^/PCNA^*−*^ quiescent fields (QF) (Supplementary Fig. S4b,c). The PF surrounds a central lumen composed of basal radial-glial-like (RG) cells with apical centrosomes and cilia that point towards the lumen (Fig. 5a,b). The nuclei of RG cells were surrounded by cage-like structures that consisted of MT. Mitotic chromosomes were frequently identified near the lumen, which suggests nuclear migration is indeed coordinated with cell cycle progression, as indicated in the neonatal brain *in vivo* (Fig. 5a). Similar to the mouse cerebral cortex in which p80 is expressed near the ventricles^18^, p80 signals were arranged around the lumen in a similar pattern to pericentrin (Fig. 5c), which suggests that p80 is localized to the centrosomes of RG cells. The QF primarily consists of neurons, astrocyte-like cells and myelin-producing cells, which originate from the PF. Consistent with the role of p80 in the proper proliferation and migration of neurons, the mutant mini-brains contained fewer TuJ1^+^ neurons than the controls, and these neurons failed to migrate out of the PF (Fig. 5d). These findings closely mirror the brain malformations identified in *KATNB1*-deficient microlissencephalic patients.

### p80, NuMA and cytoplasmic dynein form aster-like structures *in vitro*

**Figure 6:**
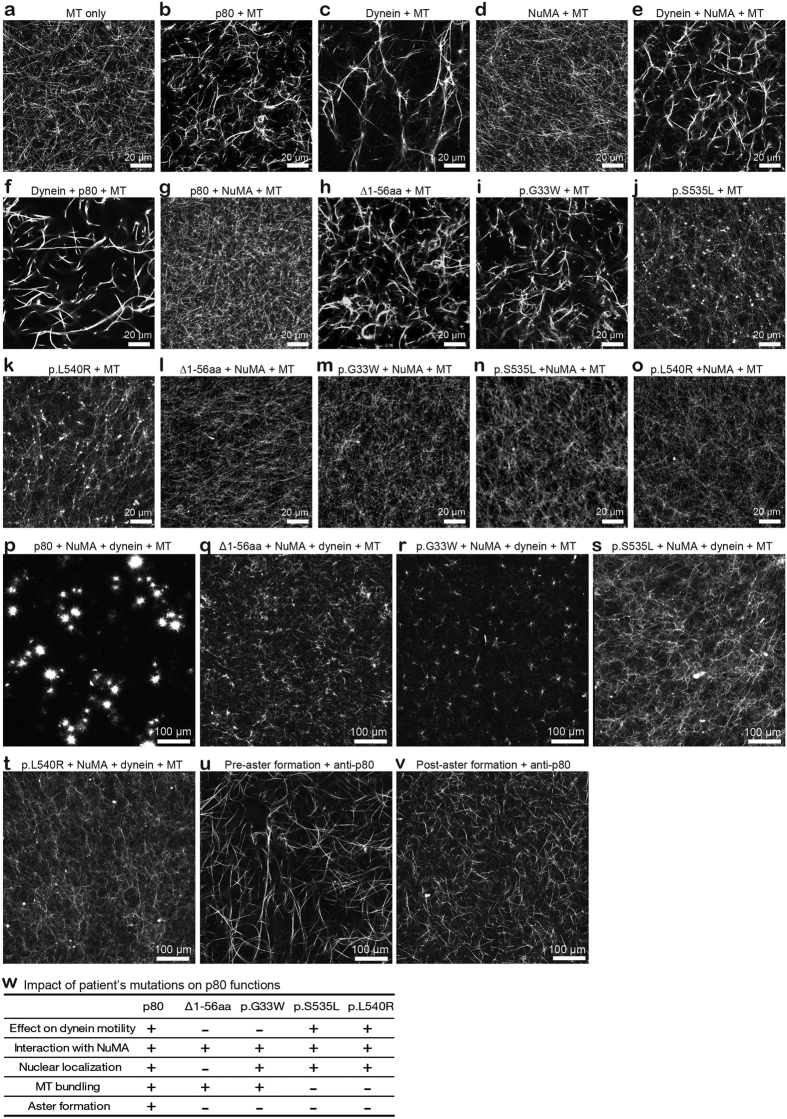
Self-organization of aster mimic structures by p80, NuMA and cytoplasmic dynein. (**a**) ATTO-565 labeled MTs in BRB80. (**b,c**) Bundling of MTs by p80 (**b**) and dynein (**c**). Addition of p80 or dynein produced a remarkable MT-bundling effect. (**d**) NuMA has no effect on MT bundling. (**e,f**) Combination of dynein and NuMA (**e**) or dynein and p80 (**f**) had no effect on MT bundling. (**g**) p80 bundling effect completely abolished by the addition of NuMA. (**h,i**) MT-bundling effect of Δ1-56 aa and p.G33W. p80 N-terminal mutation forms Δ1-56 aa (**h**) and p.G33W (**i**) both maintain MT-bundling effect. (**j,k**) Effect of p.S535 L and p.L540R on MT bundling. Two C-terminal mutations of p80 form numerous tiny assemblers when they interact with MTs. (**l–o**) Four p80 mutation forms interact with NuMA. (**p**) Robust aster formations by p80, NuMA and dynein. (**q–t**) Four p80 mutation forms have no aster formation ability despite the addition of NuMA and dynein. (**u**) Anti-p80 antibody was added after aster formation as indicated in Fig. 6p, and the asters formed by p80, NuMA and cytoplasmic dynein broke completely. (**v**) Antibody against p80 that was mixed with p80, NuMA and cytoplasmic dynein exhibited only an MT-bundling effect. All experiments were performed in 1 mM ATP conditions. Scale bar in a-o, 20 μm, and p-v, 100 μm. (**w**) Summarized table on regulatory functions for wild type p80 and its mutation forms derived from microlissencephalic patients.

NuMA was initially identified as an abundant component of interphase nuclei; with its partner, cytoplasmic dynein, NuMA tethers MTs to spindle poles^22^. Here, we determined that p80 directly interacts with NuMA and both proteins exhibit similar cellular localization throughout the cell cycle (Fig. 2a). In addition, siRNA-mediated depletion of p80 and NuMA in MEFs and mouse embryos (Figs 3 and 4 and Supplementary Fig. S2) led to similar defects in the cell cycle and cell migration. Based on these findings, we hypothesized that p80, NuMA and dynein control mitotic cell division and neuronal migration via the regulation of MT assembly at the centrosome/spindle pole. To investigate the potential function of p80, NuMA and dynein in MT assembly around the centrosome/spindle pole, we explored the direct effect of recombinant p80 and NuMA on MT organization. Compared with the untreated MTs (Fig. 6a), the addition of p80 (Fig. 6b) or dynein (Fig. 6c) exhibited remarkable MT-bundling effects, whereas the addition of NuMA (Fig. 6d) alone did not exhibit an effect on MT behaviors. Interestingly, the MT-bundling effect of p80 was completely abolished in the presence of NuMA (Fig. 6b,g) in contrast to dynein (Fig. 6e,f), which suggests that NuMA and MTs compete for binding to p80.

We subsequently investigated the effect of mutated p80 on MT bundling. Despite the loss of interaction with cytoplasmic dynein, the p80 N-terminal mutant forms Δ1-56 aa (Fig. 6h) and p.G33W (Fig. 6i) preserved the MT bundling potential. Curiously, the p.S535L and p.L540R p80 mutant proteins failed to form robust MT bundles, and instead formed numerous small piles of MT (Fig. 6j,k). As indicated with wild-type p80, NuMA abolished MT interactions regardless of the p80 mutation (Fig. 6l–o). Strikingly, robust aster formation was identified when p80, NuMA and cytoplasmic dynein were combined together in an ATP-dependent manner (Fig. 6p, Supplementary Fig. S5a,b and Supplementary Video 15), which indicates that the combination of p80, NuMA and dynein is sufficient and indispensable to organize MT into asters *in vitro*.

We subsequently investigated the effects of mutated p80 on aster formation. In contrast to the wild-type p80, none of the p80 mutant proteins (Fig. 6q–t and w) including the C-terminal truncated p80 1–314 aa (Supplementary Fig. S5c–e), formed asters in the presence of dynein, NuMA and ATP, which suggest that p80 interaction with both dynein and MT are essential to this function. Finally, we investigated whether p80 is essential for aster maintenance. An anti-p80 antibody was added to pre-formed asters following incubation with p80, NuMA and cytoplasmic dynein (Fig. 6p). As expected, asters were not formed when p80 was inactivated by anti-p80 antibody prior to aster formation (Fig. 6u). Importantly, asters formed by the triple combination of p80, NuMA and cytoplasmic dynein (Fig. 6p), were completely dissolved by the addition of anti-p80 antibody (Fig. 6v), which implies that p80 is essential for aster maintenance *in vitro*. These results provide the biochemical basis for the previously described effects of mutant p80 on the cell cycle and cell migration. Taken together, these findings suggest that p80 likely works cooperatively with NuMA and cytoplasmic dynein to promote proper MT organization at the centrosome/spindle pole during mitotic cell division and neuronal migration.

## Discussion

The current study combined *in vitro* and *in vivo* strategies to provide evidence that p80 interacts with NuMA and cytoplasmic dynein to regulate and maintain proper MT organization into asters. In contrast, four p80 mutations from microlissencephalic patients lacked these properties. These findings provide a plausible mechanism to explain the spindle abnormalities, reduced proliferation potential, increased apoptosis and reduced migration potential induced by the loss of p80 *in vitro* and *in utero*. Our findings indicate that p80 has important roles in brain development beyond its traditional role in the regulation of MT severing activity.

Similar to other autosomal recessive primary microcephaly (MCPH) genes, mutant *KATNB1* leads to a high proportion of cells with multiple centrosomes, abnormal spindle structures, and a disrupted cell cycle^18^. However, in contrast to MCPH genes, such as *STIL, CEP152, CEP135* and *CENPJ* which participate in centriole biogenesis^30^, the localization of p80 in the mitotic spindle region suggests that similar to NuMA, it participates in mitotic spindle assembly rather than directly via centriole duplication. In accordance with this hypothesis, p80-mutant iPSCs appear to form bipolar spindles with two separated centrosomes; however, they are occasionally unable to maintain the bipolar spindles, which gradually collapse into monoastral spindles. Importantly, the knockdown of NuMA has been reported to result in a similar “spindle collapse”^27^. Thus, the loss of p80 or NuMA induces cellular phenotypes that are common with other MCPHs; however, the molecular mechanisms that lead to these events may be distinct. We also show that the combination of p80, NuMA and dynein is sufficient to organize and maintain MT asters *in vitro*, which implies that these factors are involved in the maintenance of spindle architecture in mitotic cells. Direct evidence in a cellular context is still required; however, these results provide a potential explanation for spindle abnormalities in patient cells.

Here, we show for the first time that p80 and NuMA interact with each other, exhibit similar cellular localizations during the cell cycle and exhibit common phenotypes *in vivo* during mouse development. The depletion of NuMA or p80 in developing mouse embryos increased cell cycle exit and induced premature neural differentiation at the expense of stem cell expansion. These effects were particularly visible in dual-depleted brains. In addition, siRNA-mediated depletions in MEFs induced similar cytokinesis defects, which suggests that p80 and NuMA may act in a common pathway during neuronal stem cell expansion. Consistently, most patient-derived iPSCs that completed mitosis exhibited a prolonged metaphase, which was likely a result of a functional spindle assembly checkpoint that delays mitosis to enable more time for proper bipolar spindle formation. Apoptosis was identified in patient-derived iPSCs, which suggests that mitotic errors accumulate in these cells. These findings strongly suggest an important function of p80 in neuronal proliferation and survival, and the loss of p80 function may severely affect neurogenesis and ultimately result in congenital microcephaly.

p80 is a centrosome/spindle pole targeting protein^17^,^31^. Surprisingly, we identified both cytoplasmic and nuclear localization of p80 in interphase HeLa, MEF and migrating cerebellar granular cells. Here, we also demonstrate that the N-terminal region of p80 is required for its nuclear localization. Importantly, p80 localization in the nucleus is consistent with its direct interaction with NuMA. The functional roles of nuclear p80 and NuMA during interphase are predominately unknown; however, our findings that p80 and NuMA co-localize synchronously through the cell cycle in HeLa cells provide a valuable stepping stone for future studies. Interestingly, there are several exceptions. For example, p80 was determined to be centrosomal throughout the entire cell cycle in RG cells in minibrains. We have also reported that p80 exhibits a punctate centrosome distribution in E15.5 migrating neurons^23^. These cell specific localizations suggest cell-specific functions for p80. Given the importance of p80 in normal brain architecture, it is likely that centrosomal p80 has a role in neuronal migration.

The centrosome, a well-established MT organizing center, is involved not only in cell division, but also in cell migration and differentiation^26^,^32^,^33^. In migrating neurons, dynein and LIS1 activities are important for centrosome movement to the leading process in front of the nucleus, followed by nucleokinesis towards the centrosome^26^,^34^. Neuronal migration also requires optimal MT dynamics^35^,^36^,^37^. Accordingly, mutations in *LIS1* and *DCX5* cause lissencephaly, which indicates that these two genes have an important role in the stabilization of MT-dependent bridging between the nucleus and the centrosome during neuronal migration^26^. In E15.5 mouse migrating cortical neurons, Lis1 and Dcx5 form a complex with Nudel, dynein and Katanin p60 and p80^17^,^19^,^23^,^31^, presumably at the centrosomal region. Patients with homozygous *KATNB1* mutations exhibit both MCPH and lissencephaly, a condition referred to as microlissencephaly^18^,^19^, which implies that there is a common regulatory pathway between neurogenesis and neuronal migration. Consistently, patient-derived NPCs also exhibited impaired cell migration. Furthermore, iPSCs-differentiated 3D mini-brains that carried a *KATNB1* mutation consisted of fewer neurons compared to the control and exhibited substantial structural abnormalities. Strikingly, similar to LIS1, p80 directly regulates dynein motility, which suggests common mechanisms for the regulation of neuronal migration that are likely regulated by nucleus-centrosome (N-C) coupling with dynein^26^,^34^. It is likely that p80-dependent remodeling and tethering of MTs is essential for nucleokinesis in migrating neurons. An attractive hypothesis is that during nucleokinesis, dynein-p80-NuMA stabilize MT-dependent bridging between the nucleus and centrosome, potentially as part of a larger complex that includes LIS1-DCX-NUDEL-P60.

NuMA is a large protein that is thought to bundle MTs together and focus the MT minus-ends at the centrosomes during mitosis^38^,^39^. Furthermore, a cortical protein complex, including LGN, NuMA, and dynein/dynactin, plays a key role in the establishment of proper spindle orientation during asymmetric divisions^40^. Similar to many other MT organizing proteins (e.g., lamin-A/C, LAP2α and BAF1^41^), NuMA shuttles between the nucleus and the spindle poles in synchrony with the cell cycle in HeLa cells: NuMA is localized in the nucleus during interphase, on the spindle pole during metaphase and with DNA at anaphase. Here, we show for the first time that NuMA also has a role in neuronal migration. The *in vivo* depletion of p80 or NuMA in developing mouse embryos led to apparent defects in neuronal migration, particularly in dual-depleted brains. These shared phenotypes support the notion of a common regulatory pathway for NuMA and p80. Thus, our finding raises the question: how does NuMA, which is localized in the nucleus during interphase, drive neuronal migration? One potential explanation is based on the finding that NuMA localization is cell specific during interphase. NuMA is expressed in the nucleus in interphase iPSCs, fibroblasts and RG cells, whereas it appears as multiple small particles within the somatodendritic compartment of neurons, where its levels increase during early dendritic differentiation^42^. Here, we show that NuMA is expressed in the perinuclear region and nuclei in migrating granular cells, whereas post-mitotic neurons exhibited NuMA in both the nucleus and in the cytoplasm. Interestingly, two NuMA isoforms, which are likely to be expressed at low levels, were found to be clustered at the centrosomal region in interphase CHOP cells^43^. These findings suggest the possibility that, similar to p80, cytoplasmic NuMA may participate in neuronal migration.

The scattering of p80 from the mitotic poles after nocodazole treatment indicates that similar to NuMA, p80 is a part of the mitotic spindle rather than an integral part of the centrosome. Therefore, p80 is more likely to regulate MT dynamics rather than participate in centriole biogenesis. In support of this idea, we determined that a p80-NuMA-dynein-MT complex is sufficient to form asters *in vitro* in the presence of ATP. We also show that patient mutations are unable to bind to dynein (i.e., mutations in the N-terminal of p80) or to bundle MTs (i.e., mutations in the C-terminal of p80); as a consequence, they are unable to elicit aster formation. Our finding that p80 directly regulates dynein motility, suggests that p80 modulates spindle assembly by mobilizing proteins to the centrosome. In the absence of functional p80, several proteins, including p60, LIS1 and dynein were partially mislocalized from the centrosome^18^,^19^. Nevertheless, our finding that the inactivation of p80 through the addition of anti-p80 antibodies dissolved pre-formed asters, suggests that p80 is also important for aster maintenance when it is in a complex with NuMA, dynein and MTs. We and other researchers have found that p80 carries the ability to bundle MT *in vitro*^17^. Interestingly, NuMA abrogated the MT-bundling effect of p80, which suggests that NuMA and MT bind to p80 in a competitive manner. Thus, p80 may not be in direct contact with MT when in complex with NuMA; however, it may interact with MTs in other contexts, e.g., severing activity with p60. Future studies will aim to identify additional components of this complex and how this complex functions in the context of neuronal differentiation and migration.

In conclusion, our findings that a p80-NuMA-dynein-MT complex is sufficient to form asters *in vitro*, whereas p80 mutations from patients were unable to elicit aster formation, indicate that severe brain abnormalities in patients may be, in part, a result of the loss of the ability of p80 to regulate MT dynamics. These findings provide novel molecular and cellular insights into the potential pathogenesis of severe microlissencephaly.

## Methods

The methods were conducted in accordance with the relevant guidelines. All experimental protocols were approved by the Institution’s Gene Modification Experiments Safety Committee of Osaka City University (authorized number; OCU-142601) and Singapore (IRB-10051). All mouse experiments were performed with the approval of the Institution’s Animal Care and Ethics Committee of Osaka City University (authorized number; OCU-08033).

### Plasmids and recombinant proteins

The NuMA construct was a generous gift from Dr. Duane Compton, whereas p80 C-terminal constructs that contained the S535L and L540R mutations were generous gifts from Dr. Murat Gunel. The wild-type, truncated and mutated p80 were subsequently sub-cloned into a pEGFP vector (Clontech Laboratories, CA, USA). Recombinant p80 and NuMA^44^ proteins were generated using a bacterial expression system (Invitrogen) and pGEX-4T expression vector (GE Healthcare Lifesciences, UK). Protein purification was performed using Glutathione Sepharose 4B (GE Healthcare Lifesciences, UK) according to the manufacturer’s instructions. The glutathione S-transferase (GST) tag was removed from recombinant proteins by thrombin treatment (GE Healthcare Lifesciences, UK).

### Protein Purification

Porcine brain cytoplasmic dynein and tubulin were purified as previously described^45^. To prepare fluorescently labeled MTs, tubulin was labeled with TMR dye (FluoReporter TMR Protein Labeling Kit; Thermo Fisher Scientific, MA, USA) or ATTO565 or ATTO647N dye (ATTO-tech, Siegen, Germany) according to the manufacturer’s labeling protocol.

### Culture of human cells

Human iPSCs were generated as previously described^18^. iPSCs were dissociated to form cell aggregates and induced to form NSCs in neural induction medium (DMEM/F12 supplemented with 20% KOSR, 2 mM L-glutamine, 0.2 mM NEAA, 0.1 mM 2-mercaptoethanol and 1 mM sodium pyruvate) for 7 days. The clumps of cells were adhered onto Matrigel-coated dishes in neural precursor medium (Neurobasal medium supplemented with 2 mM L-glutamine, 1% B27, 1% N2 and 20 ng/ml bFGF) for the formation of rosette-like structures for an additional 7–14 days. NPCs were plated on Matrigel-coated dishes for live cell imaging.

### Immunoprecipitation (IP) assay

IP experiments were performed as previously described^25^ with several modifications. Anti-GFP or anti-p80 antibodies were cross-linked with protein G-Sepharose beads (GE Healthcare Lifesciences, UK) for 2 h at 4 °C. GFP or three GFP-fusion p80 constructs were overexpressed in MEF cells. The cells were resuspended in a homogenization buffer (20 mM Tris, pH 8.0, 0.1 M KCl, 1 mM PMSF, 5 μg/ml each of aprotinin, leupeptin and pepstatin A, and 0.2% Triton X-100). The supernatants from the cell lysates or mouse brain extracts were subsequently harvested via centrifugation. IP analyses were performed for 2 h using anti-GFP antibody (JL-8, BD Biosciences, SJ, California) or anti-p80 antibody^23^, respectively. After three washes, the complexes were eluted in SDS-PAGE sample buffer or 0.1 M Glycine buffer (pH 2.8)/1 M Tris (pH 10) and analyzed via WB or LC-MS/MS.

### *In vitro* MT-gliding assay

A quantity of 0.4 μM dynein was added to a 5 mg/ml BSA precoated flow chamber (Matsunami Glass, Osaka, Japan) and adsorbed onto the bottom for 5 min. A total of 10.5 mg/ml TMR-labeled tubulin and 7.64 mg/ml white tubulin were polymerized at a ratio of 1:40 and stabilized in 50 μM Taxol (Sigma)/BRB80 buffer (40 mM PIPES, pH 7.2, 0.5 mM MgSO_4_, and 0.5 mM EGTA). The MT solution was diluted to 0.5 μM in ATP buffer (1 mM ATP and 100 μM Taxol) with oxygen scavenger (225 μg/ml glucose, 216 μg/ml glucose oxidase, 36 μg/ml catalase, and 1% 2-mercaptoethanol) and introduced into the flow chamber. Following confirmation of MT gliding by cytoplasmic dynein (0.5 μM), recombinant proteins (p80; 0.4 μM, Δ1-56 aa; 0.4 μM, p.G33W; 0.4 μM, p.S535 L; 0.4 μM, p.L540R; and 0.4 μM, BRB80) were added. MT gliding was observed via conventional inverted fluorescence microscopy (Olympus IX71, Tokyo, Japan) with an oil-immersion objective lens (UPlanSAPO, 100X, NA = 1.4, Olympus, Tokyo, Japan) and an EMCCD camera (ImagEM, Hamamatsu Photonics, Hamamatsu, Japan). Captured images were analyzed with AquaCosmos software (Hamamatsu Photonics, Hamamatsu, Japan). All experiments were performed at 37 °C with a stage incubator (Tokai-Hit, Shizuoka, Japan).

### Synchronization of HeLa cells

HeLa cells were cultured in DMEM supplemented with 10% FCS. For mitosis synchronization, sub-confluent HeLa cells were incubated with 2 mM thymidine for 16 h prior to washing with PBS and subsequently released into normal growth medium for 9 h. Two mM thymidine were added for an additional 16 h, and the cells were released immediately prior to the S phase of the cell cycle.

### Immunocytochemistry

MEF or HeLa cells were fixed with 4% (w/v) ultra-pure electron microscopy-grade paraformaldehyde for 20 min at 37 °C and permeabilized with 0.2% Triton X-100 for 10 min at room temperature. The cells were subsequently blocked with 5% (w/v) BSA and Block Ace Powder (DS Pharma Biomedical Co., Ltd., Osaka, Japan) in PBS for 1 h at room temperature, followed by incubation with a combination of 2 of the following antibodies for 1 h at room temperature: anti-p80 antibody^23^, anti-NuMA antibody (Bethyl Laboratories, Montgomery, USA), anti-γ tubulin antibody (Sigma), and anti-β tubulin antibody (Abcam). The samples were washed off with PBS, and subsequently incubated with Alexa 488-conjugated anti-mouse IgG or Alexa 546-conjugated anti-rabbit IgG (Thermo Fisher Scientific, MA, USA) that contained 400 nM 4′,6-diamidino-2-phenylindole (DAPI) for 1 h at room temperature. Slides were mounted in FluoSave Reagent (EMD Millipore, Darmstadt, Germany), and images were obtained with a laser scanning confocal microscope (LSM700, Carl Zeiss, Oberkochen, Germany).

### 3D alignment measurements

Cells were stained with anti-NuMA (A301-510A, Bethyl), anti-pericentrin (AB4448, Abcam) and anti-p80 (LS-C173437, LS Bio) antibodies. Images of cells in metaphase were obtained using a Delta-vision RT inverted microscope (magnification 100x), followed by deconvolution. 3D alignment measurements were calculated using Imaris software (Bitplane, Version 7.7.2). The Pearson’s coefficient in the ROI volume was calculated for each double staining.

### *In vitro* aster formation using recombinant and purified proteins

ATTO647- and ATTO565-MTs were polymerized for 30 min at 37 °C and stabilized with 40 μM paclitaxel (Sigma). The labeling ratios were approximately 15%. Purified cytoplasmic dynein (0.4 μM) was mixed with MTs and different combinations of recombinant proteins (p80, 0.4 μM; NuMA, 0.4 μM; Δ1-56 aa, 0.4 μM; p.G33W, 0.4 μM; p.S535 L, 0.4 μM; or p.L540R, 0.4 μM). Following incubation at 37 °C for 10 min, the samples were loaded into an observation chamber composed of two cover glasses (32 mm × 24 mm and 18 mm × 24 mm) precoated with Teflon (Furuta *et al*., in preparation) and spaced 100 μm from each other with silicon rubber (Asone). The chamber was passivated with 1% (w/v) Pluronic F127/BRB80 for 10 min and subsequently loaded with a mixed solution of MTs/p80/NuMA/dynein in ATP buffer as previously described. Images were obtained with a confocal laser-scanning microscope system (Nikon A1 and Ti-E) using a Plan Apo Lambda 40X Ph2 DM (NA = 0.95, Nikon) objective lens.

### Small interfering RNA and cell culture

siRNAs targeted to the sequence of mouse katanin p80: GCUGCAGAGCAAGUAUGAGAGCUAU, mouse NuMA: GCUUCAAGUAGAAACAGCCAGCAAU and firefly (*Photinus pyralis*) luciferase (pGL2): CGUACGCGGAAUACUUCGATT as a negative control were synthesized by Thermo Fisher (MA, USA) or Sigma-Aldrich (MO, USA). Each double-stranded 25- or 21-nucleotide RNA at a final concentration of 200–400 nM was transfected into MEF cells using a Neon transfection system (Thermo Fisher Scientific, MA, USA). The MEF cells were plated onto poly-L-lysine-coated 6-well plates (IWAKI Glass Inc., Tokyo, Japan) and cultured in DMEM (Wako Chemicals, Osaka, Japan) with 10% heat-inactivated fetal bovine serum (Nichirei Biosciences, Tokyo, Japan) for 48 h prior to analysis. For the rescue experiments, GFP-hp80 and hNuMA-GFP constructs were co-transfected with mouse p80 and NuMA siRNA, respectively.

### Analysis of mitotic cells via live cell imaging

Cells were treated with 0.3 μM SiR-tubulin (SC006, Cytoskeleton) for 8 h, followed by 5 μg/ml Hoechst 33342 dye for 5 min. After the medium was changed to fresh growth medium, live cell imaging was performed using an Olympus IX-83 microscope. The time between prophase and anaphase was measured in cells that entered mitosis during live cell imaging.

### *In utero* electroporation

*In utero* electroporation was performed using pregnant ICR mice as previously described^46^, with minor modifications. Approximately 1 μl of the plasmid solution (0.3 μg pCAG-GFP and 200 nM target siRNA with 0.03% fast green) was injected into the lateral ventricle of intrauterine embryos. The head of each embryo was placed between the disks of a forceps-type electrode (3 mm disk electrodes, CUY650P3; NEPA GENE, Chiba, Japan), and genes were electroporated into the cerebral walls by electronic pulses (30–50 V, 50 ms, five times).

### Preparation of frozen sections

The mouse embryos were placed on ice with or without transcardial perfusion using a periodate-lysine-paraformaldehyde (PLP) fixative, pH 7.4. After decapitation, embryonic brains were collected and fixed in the same fixative for at least 2 h at 4 °C. Following fixation, the brains were placed in a 20% sucrose solution and subsequently embedded in OCT compound (Sakura). The samples were stored in cryogenic conditions and cut with a cryostat into 16-μm-thick sections.

### Immunohistochemistry

Brain tissue sections were blocked with 3 mg/ml BSA/PBS that contained 0.1% Triton X-100 (Wako). Primary antibodies were applied overnight at 4 °C (anti-Ki67 [Novocastra, Tokyo, Japan] at 1:100 dilution; anti-PH3 [EMD Millipore, Darmstadt, Germany] at 1:250; anti-GFP [Aves, Oregon, USA] at 1:1000; anti-P27 [BD Biosciences] at 1:500). The sections were washed 3 times with PBS and incubated with Alexa 488-, Alexa 546-, and Alexa 647-conjugated secondary antibodies (Thermo Fisher), respectively, for 1.5 h at room temperature. Fluorescence images were obtained using a confocal microscope (FV1000, Olympus).

### Mini-brain differentiation

Mini-brains were generated as described in Lancaster and Knoblich, 2013^29^. Staining was performed using antibodies against p80 [LS-C173437, LS Bio], pericentrin [AB4448, Abcam], alpha tubulin [DM1A, Abcam], PCNA [ab 18197], and acetylated tubulin [T6793, Sigma].

## Additional Information

**How to cite this article**: Jin, M. *et al*. Katanin p80, NuMA and cytoplasmic dynein cooperate to control microtubule dynamics. *Sci. Rep.* **7**, 39902; doi: 10.1038/srep39902 (2017).

**Publisher's note:** Springer Nature remains neutral with regard to jurisdictional claims in published maps and institutional affiliations.

## Supplementary Material

Supplementary Movie 1

Supplementary Movie 2

Supplementary Movie 3

Supplementary Movie 4

Supplementary Movie 5

Supplementary Movie 6

Supplementary Movie 7

Supplementary Movie 8

Supplementary Movie 9

Supplementary Movie 10

Supplementary Movie 11

Supplementary Movie 12

Supplementary Movie 13

Supplementary Movie 14

Supplementary Movie 15

Supplementary Figures
