# Supplementary material for: Katanin p80, NuMA and cytoplasmic dynein cooperate to control microtubule dynamics
Source: Sci Rep. 2017 Jan 12;7:39902. doi: 10.1038/srep39902 (PMC5228124; doi:10.1038/srep39902)

Supplementary Information

Katanin p80, NuMA and cytoplasmic dynein cooperate to control microtubule dynamics

Mingyue Jin[1,*], Oz Pomp[2,*], Tomoyasu Shinoda[5], Shiori Toba[1], Takayuki Torisawa[6,7], Ken'ya Furuta[6,7], Kazuhiro Oiwa[6,7,8], Takuo Yasunaga[9,10,11], Daiju Kitagawa[12], Shigeru Matsumura[13], Takaki Miyata[5], Thong Teck Tan[2], Bruno Reversade[2,3,4,#] and Shinji Hirotsune[1,#]

1. Department of Genetic Disease Research, Osaka City University, Graduate School of Medicine, Asahi-machi 1-4-3, Abeno, Osaka 545-8585, Japan
2. Institute of Medical Biology, Human Genetics and Embryology Laboratory, Singapore
3. Institute of Molecular and Cellular Biology, A*STAR, Singapore 138648, Singapore
4. Department of Paediatrics, National University of Singapore, Singapore 119260, Singapore
5. Department of Anatomy and Cell Biology, Nagoya University Graduate School of Medicine, 65 Tsurumai, Showa, Nagoya 466-8550, Japan
6. Advanced ICT Research Institute, National Institute of Information and Communications Technology, Kobe, Hyogo 651-2492, Japan
7. CREST, Japan Science and Technology Agency, Chiyoda-ku, Tokyo 102-0076, Japan
8. Graduate School of Life Science, University of Hyogo, Harima Science Park City,

Hyogo 678-1297, Japan

9. Department of Bioscience and Bioinformatics, Faculty of Computer Science and Systems Engineering, Kyushu Institute of Technology, Kawazu 680-4, Iizuka, Fukuoka 820-850, Japan
10. JST-SENTAN, 4-1-8, Honcho, Kawaguchi, Saitama 332-0012, Japan
11. JST-CREST, 4-1-8, Honcho, Kawaguchi, Saitama 332-0012, Japan
12. Division of Centrosome Biology, Department of Molecular Genetics, National Institute of Genetics, 1111 Yata, Mishima, Shizuoka, 411-8540, Japan
13. Department of Cell Biology, Institute for Virus Research, Kyoto University, Sakyo-ku, Kyoto, 606-8507, Japan

*: These authors contributed equally to this work.

#: Co-senior author

Correspondence should be addressed to: Bruno Reversade or Shinji Hirotsune

Bruno Reversade:

Tel: (+65) 8282 5711

Fax: (+65) 6464 2006

e-mail: bruno@reversade.com

Shinji Hirotsune:

Tel: +81-6-6645-3725

Fax: +81-6-6645-3727

e-mail: shinjih@med.osaka-cu.ac.jp

Supplementary Figure Legends

Supplementary Figure S1. P80 co-localized with NuMA. (**a**) To identify new p80 interaction partners, immunoprecipitation was performed with anti-p80 antibody [23] and mouse embryonic brain lysate. Co-precipitated proteins (left) were treated with trypsin to generate peptides. The resulting tryptic peptides were fractionated with a cation exchange column using several different concentrations of KCl (10, 50, 70, 100, 200 and 350 mM). The peptide fractions were resuspended in 25 μl of 0.1% formic acid and subjected to LC-MS/MS analysis using Analyst QS Software 2.0 (AB Sciex) in the positive-ion mode. Identified peptides were analyzed with ProteinPilot using the NCBI database (RefSeq, release 61, September, 2013, http://www.ncbi.nlm.nih.gov/news/09-18-2013-refseq-release61/). The minimum threshold for protein identification was set at a protein score of 0.47, which corresponds to a confidence level greater than 66% (Supplementary Table S1). The listed proteins were identified from our analyzed results as strong candidates to interact with p80. (**b**) p80 expression in interphase HeLa cell. p80 is expressed in both the cytoplasm and nucleus. Scale bar, 10 μm. (**c**) Migrating granular cells isolated from postnatal day 3 mouse pups. Scale bar, 100 μm. (**d,e**) p80 and NuMA expression in migrating granular cells. Magenta rectangle-surrounded areas in (**d**) and (**e**) were enlarged and are presented in the lower panels. Both proteins were expressed in the cytoplasm and nuclei in migrating neurons. Scale bar, 10 μm. (**f,g**) 3D analysis of co-localization of p80 with NuMA (**f**) and pericentrin (**g**) using Imaris software. Scale bar, 2 μm. (**h,i**) The silencing effect of p80 and NuMA siRNAs in granular cells. siRNAs against mouse p80 (**f**) or NuMA (**g**) were also assessed for their effects in granular cells. Lower quantitative graphs (n = 3) indicate that siRNAs also effectively decreased p80 (**f**) or NuMA (**g**)

expression in neuronal cells. β-actin was used as a loading control. *P*-values were calculated with Student's *t*-test, mean ± s.e., *** $P < 0.001$.

Supplementary Figure S2. Defective mitosis caused by p80 or NuMA depletion in MEF cells. (**a-e**) Aberrant mitotic phenotypes of MEF cells treated with siRNAs against p80 (**a,b**), NuMA (**c,d**) or both (**e**). siRNA treated cells were stained with anti-γ tubulin antibody (green) to probe centrosomes/spindle poles and anti-β tubulin antibody (red) to probe MT skeletons. In (**a**) and (**c**), cells with the depletion of the indicated target proteins exhibited multipolar (upper panels) and monopolar (middle panels) spindles, accompanied by cytokinesis defects (lower panels). (**b**), (**d**) and (**e**) present image galleries of aberrant mitotic phenotypes as indicated. (**f**) Aberrant centrosomal duplication in p80 or NuMA siRNA treated interphase MEF cells. Rectangle-surrounded centrosomes were enlarged and are presented as insets. (**g**) Rescue of aberrant centrosomal duplication by co-transfection of NuMA siRNA with hNuMA-GFP (lower) or p80 siRNA with GFP-hp80 (upper). Rectangle-surrounded centrosomes were enlarged and are presented as insets. (**h**) Quantitative data for (**f**) and (**g**). In each case, 100 siRNA treated cells were subjected to the analysis. Indicated *P* values were calculated via ANOVA, mean ± s.e., ** $P < 0.01$, *** $P < 0.001$. Scale bar, 10 μm.

Supplementary Figure S3. Mitotic abnormalities in patient-derived iPSCs. (**a**) Collapsed mitotic spindles resulted in monoaster formation. (**b**) Staining of monoaster cell with the indicated antibodies. (**c**) Delayed metaphase in patient iPSCs (Δ1-56 aa). Prophase lengths in control and patient iPSCs were captured via live cell imaging. DNA is visualized by Hoechst; mitotic cells are indicated with red arrowheads. Quantitative

graph is presented in Fig. 3g. (**d**) Aborted cytokinesis in patient-derived iPSCs (Δ1-56 aa). (**e**) Asymmetric distribution of chromosomes during mitosis in patient-derived iPSCs (Δ1-56 aa). Yellow arrowheads indicate mitotic poles; green arrowheads indicate DNA. Scale bar, 10 μm.

Supplementary Figure S4. Staining of normal mini-brain for the indicated markers. (**a**) Patient (c. 1A>G, Δ1-56 aa) [18]-derived iPSCs differentiated into a homogeneous population of $Nestin^+$ NPCs used for cell motility (Supplementary Video 14). Scale bar, 50 μm. (**b**) Differentiated normal mini-brain (left) exhibited distinctive fields with proliferating cells (PF) or quiescent neurons (QF) (right). Scale bars, 500 μm for left image and 100 μm for right image. (**c**) PF and QF were probed with the indicated antibodies. Rectangle-surrounded areas were magnified below. Scale bar, 50 μm. PF, proliferating field; QF, quiescent field; L, lumen; MT, microtubule.

Supplementary Figure S5. Full-length p80 is essential for aster formation in an ATP-dependent manner. (**a**) Without ATP, cytoplasmic dynein also has an MT-bundling effect. (**b**) Triple combination of p80, NuMA and dynein in an ATP-absent condition completely failed to form asters. Square-surrounded area was enlarged and is presented on the right side. (**c**) p80 1-314 aa (an N-terminal WD40 repeat domain that interacts with dynein) did not interact with MTs. (**d**) Double combination of p80 1-314 aa and dynein resulted in large and irregular MT clusters. (**e**) Triple combination of p80 1-314 aa, dynein and NuMA has no ability to make aster-like structures. Scale bars, 20 μm in (**a** and **c,d**) and 100 μm in (**b** and **e**).

Supplementary Video Legends

Supplementary Video 1: *In vitro* MT gliding assay. Dynein molecules smoothly transport MTs (white) in 1 mM ATP-containing conditions. Scale bar, 10 μm.

Supplementary Video 2: Effect of p80 on dynein motility. MT-gliding activity of dynein was completely inhibited by the addition of p80. Scale bar, 10 μm.

Supplementary Video 3: Effect of p80 Δ1-56 aa on dynein motility. MT-gliding activity of dynein was not affected by p80 N-terminal truncated form Δ1-56 aa. Scale bar, 10 μm.

Supplementary Video 4: Effect of p80 p.G33W on dynein motility. MT-gliding activity of dynein was not affected by the addition of p80 point mutation p.G33W. Scale bar, 10 μm.

Supplementary Video 5: Effect of p80 p.S535L on dynein motility. MT-gliding activity of dynein was inhibited by the addition of p80 point mutation p.S535L. Scale bar, 10 μm.

Supplementary Video 6: Effect of p80 p.L540R on dynein motility. MT-gliding activity of dynein was inhibited by the addition of p80 p.L540R. Scale bar, 10 μm.

Supplementary Video 7: Normal mitotic iPSCs probed by DNA. Normal mitotic iPSCs were identified by probing DNA with Hoechst.

Supplementary Video 8: Prolonged prophase length in patient (c. 1A>G, Δ1-56 aa) [18] iPSC. DNA and MTs were visualized with Hoechst (blue) and SiR-tubulin (red), respectively.

Supplementary Video 9: Aborted cytokinesis in patient-derived iPSC. Reversal of chromosome condensation was identified in patient (c. 1A>G, Δ1-56 aa) [18] iPSCs. DNA and MTs were visualized with Hoechst (blue) and SiR-tubulin (red), respectively.

Supplementary Video 10: Spindle is collapsed into monoaster. Monoaster formation results from collapsed mitotic spindle in patient (c. 1A>G, Δ1-56 aa) [18] iPSC.

Supplementary Video 11: Control human wild type neural progenitor cells (NPCs) for investigation of apoptosis. Cell nuclei were probed with pLVX-H2B-mCherry (red), and cytoplasm was visualized with TUB GFP-Puro (green). Cells were observed for 10 h at 5-min intervals. In wild type cells, there were no detectable apoptotic cells. Scale bar, 20 μm.

Supplementary Video 12: Apoptotic cells in patient-derived NPCs. Cell nuclei and cytoplasm were probed as described in Video 7. After 4 h of observation, patient-derived cells (c. 1A>G, Δ1-56 aa) [18] began to undergo apoptosis (lower right), as demonstrated by the nuclear fragmentation. Scale bar, 20 μm.

Supplementary Video 13: *In vitro* cell motility using human wild type NPCs. Actively

moving NPCs were observed in healthy neural progenitor cells. Scale bar, 20 μm.

Supplementary Video 14: *In vitro* cell motility of patient-derived p80 depletion NPCs. Clearly impaired cell movements were identified in p80 depletion neural progenitor cells (c. 1A>G, Δ1-56 aa) [18]. Scale bar, 20 μm.

Supplementary Video 15: *In vitro* aster mimic structure constructed by p80, NuMA and dynein. p80, NuMA, and cytoplasmic dynein with ATTO-565 labeled MTs formed an aster mimic structure in an ATP-dependent manner. Scale bar, 20 μm.

Supplementary Table Legend

Supplementary Table S1. Spectra of one peptide ID in the katanin p80 interactome listed in Supplementary Fig. S1a.

Jin and Pomp et al., Supplementary Figure S1:

a

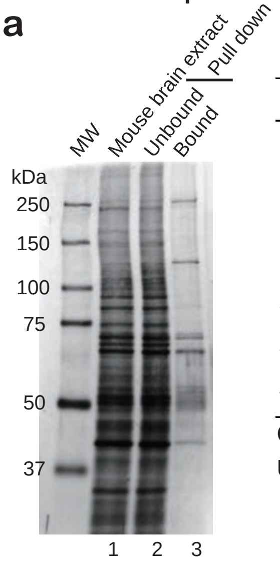

Identified proteins by LC-MS/MS

Name	% Coverage	Accession #	Unused	Total
katanin p80 WD40 repeat-containing subunit B1	62.3	gi|84095201	1.24	1.24
cytoplasmic dynein 1 heavy chain 1	80.9	gi|134288917	2.88	2.89
cytoplasmic dynein 1 light intermediate chain 2	40	gi|225543181	2.03	2.03
nuclear mitotic apparatus protein 1	56.4	gi|254675300	2.59	2.63
tubulin beta-2B chain	85.2	gi|21746161	21.47	21.47
tubulin alpha-1A chain	88	gi|6755901	23.47	23.47
tubulin beta-3 chain	86.4	gi|12963615	2.65	16.63

Coverage: Percent sequence coverage based on the presence of peptides
Unused: ProteinPilot unused score reflects the amount of total, unique peptide evidence related to a given protein.

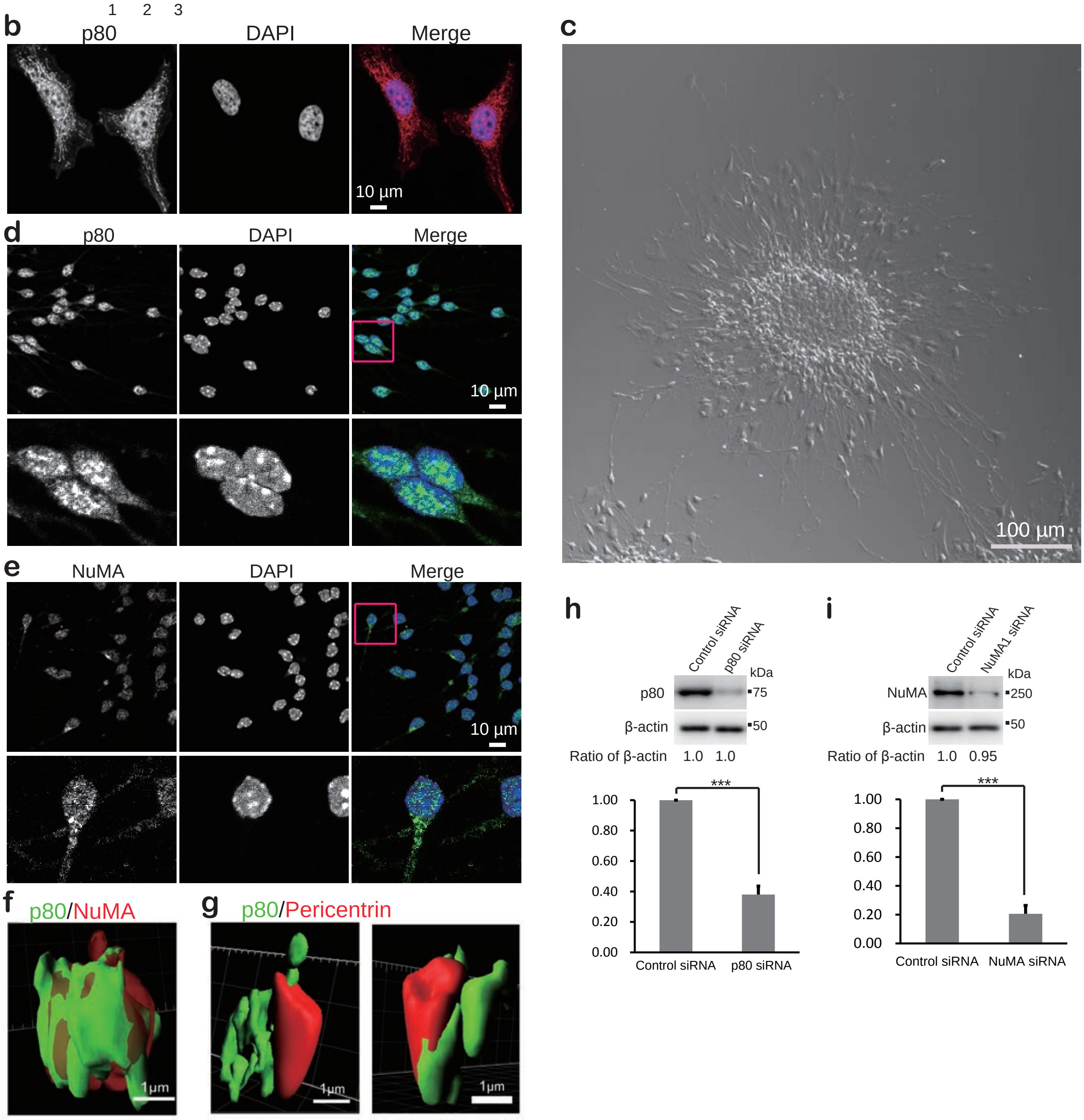

Jin and Pomp et al., Supplementary Figure S2:

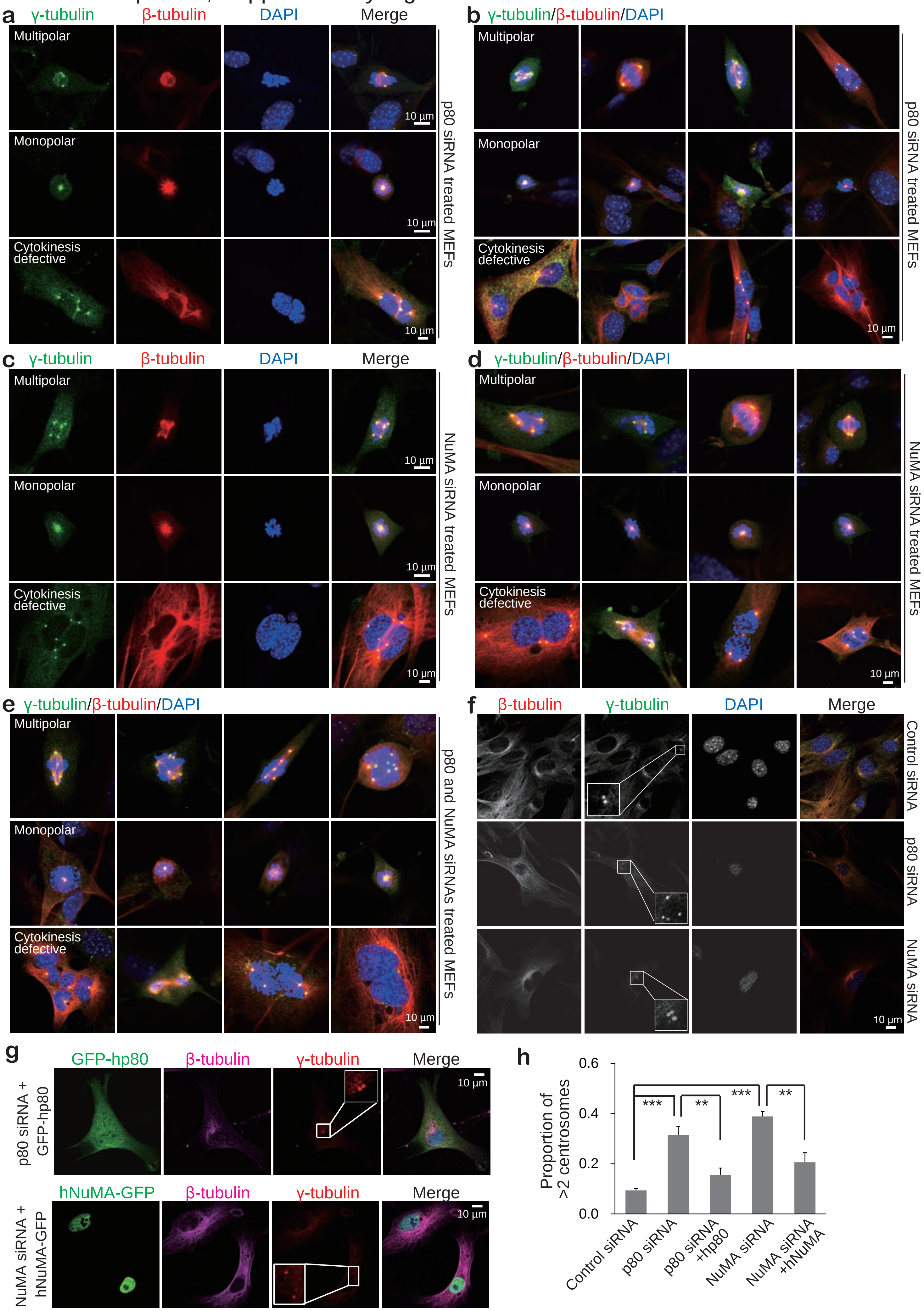

Jin and Pomp et al., Supplementary Figure S3:

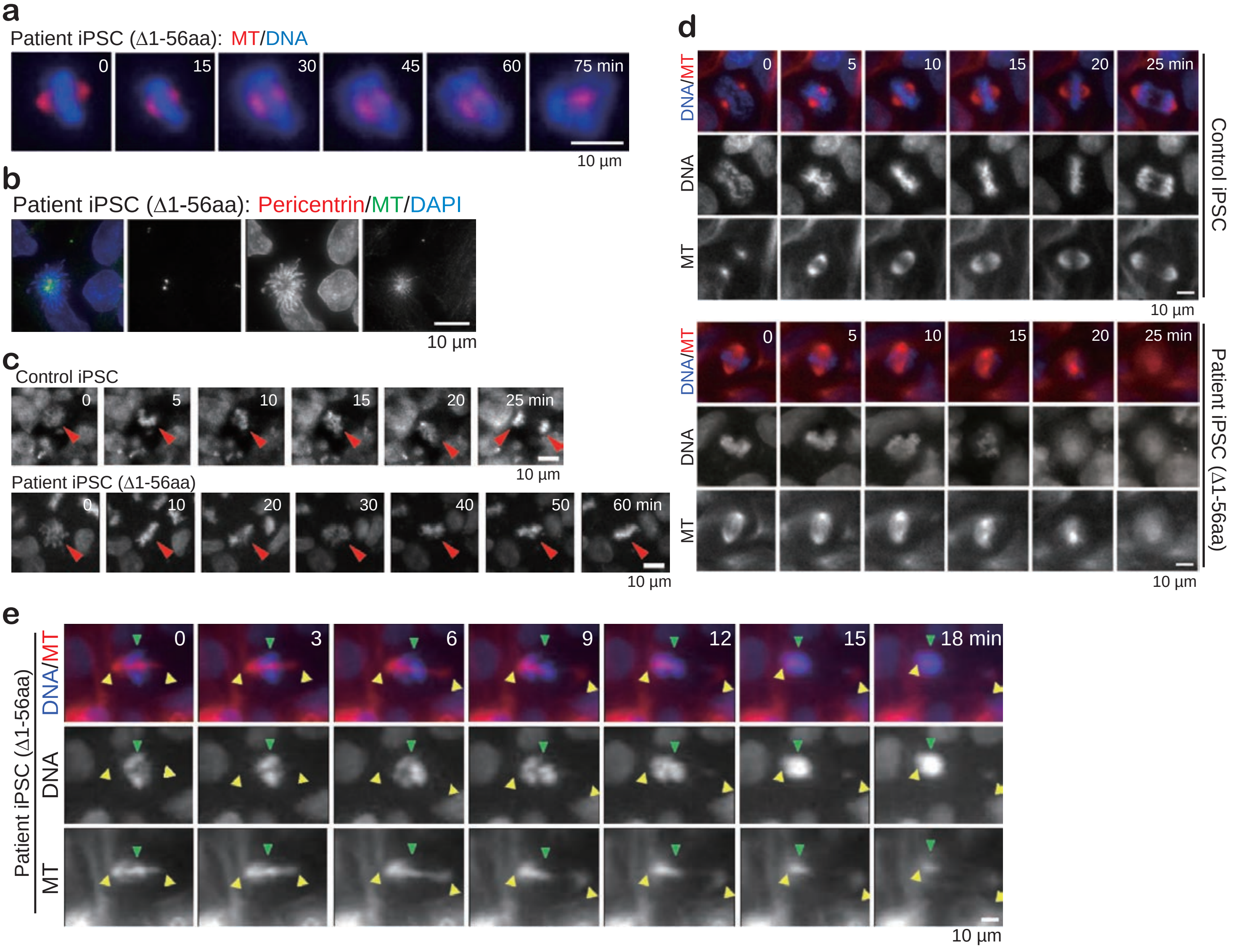

Jin and Pomp et al., Supplementary Figure S4:

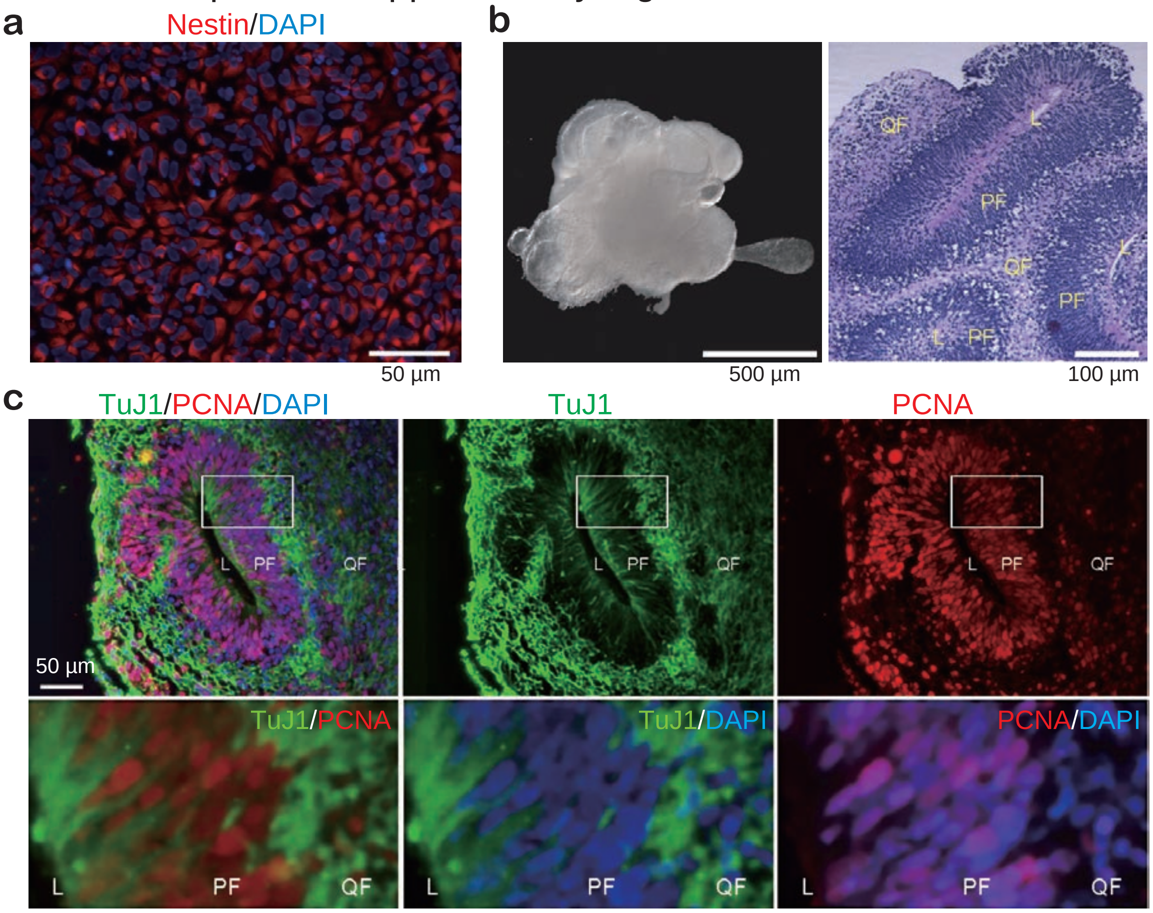

Jin and Pomp et al., Supplementary Figure S5:

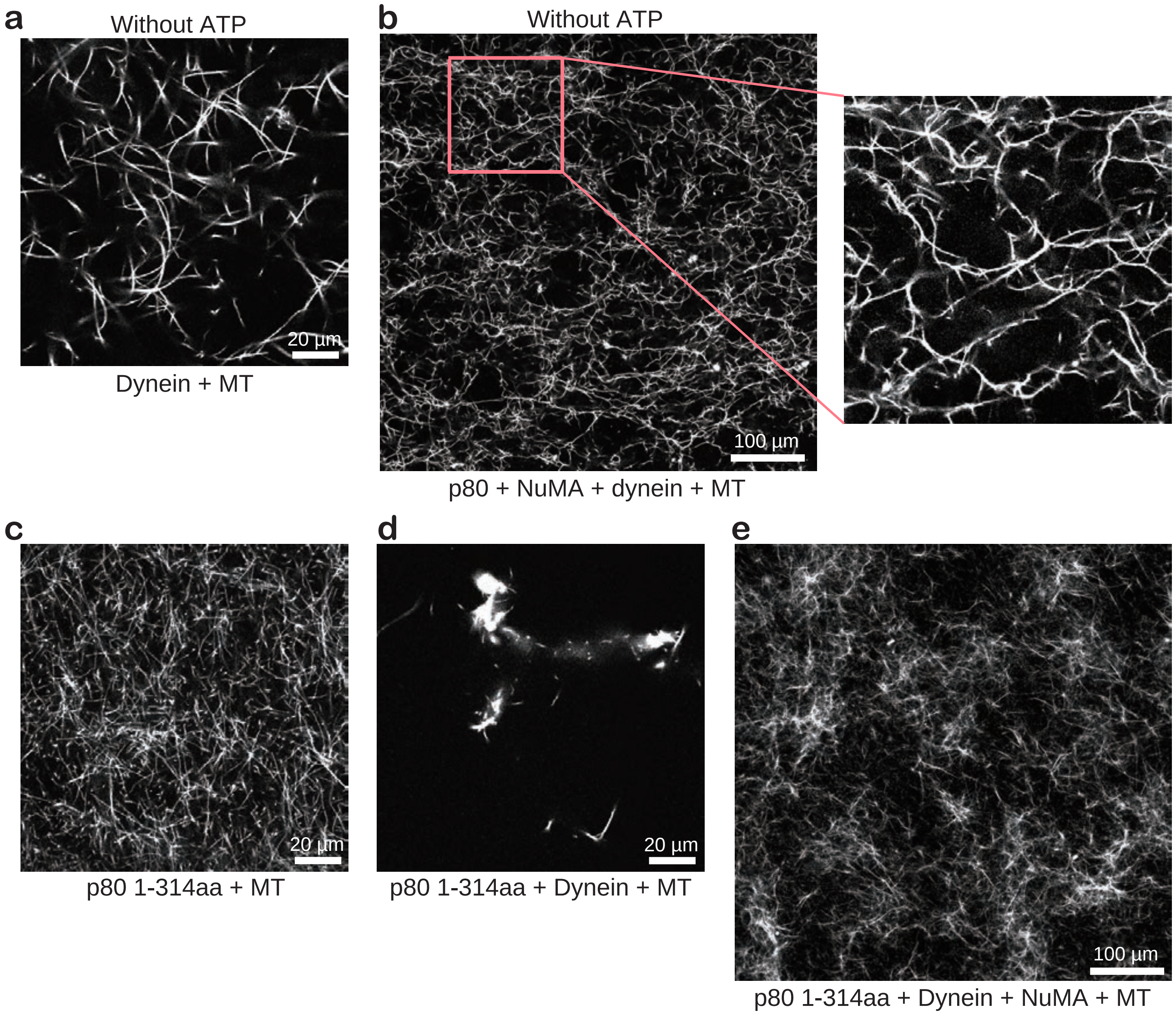

Jin and Pomp et al., Supplementary Table S1

Katanin p80

MS/MS Fragmentation of SLTGHTSPVESVR found in katanin p80 WD40 repeat-containing subunit B1

Fragmentation Evidence

Residue	b	b-H2O	b-H3PO4	b-NH3	b-OH	y	y-H2O	y-H3PO4	y-NH3
S	88.0393	70.0287		71.0128	71.0366	1369.707	1351.697	1271.73	1352.681
L	201.1234	183.1128	103.1465	184.0968	184.1206	1282.675	1264.664	1184.698	1265.648
T	302.171	284.1605	204.1942	285.1445	285.1683	1169.591	1151.58	1071.614	1152.564
G	359.1925	341.1819	261.2156	342.166	342.1898	1068.543	1050.533	970.5664	1051.517
H	496.2514	478.2409	398.2745	479.2249	479.2487	1011.522	993.5112	913.5449	994.4952
T	597.2991	579.2885	499.3222	580.2726	580.2964	874.4629	856.4523	776.486	857.4363
S	684.3311	666.3206	586.3542	667.3046	667.3284	773.4152	755.4046	675.4383	756.3886
P	781.3839	763.3733	683.407	764.3573	764.3812	686.3832	668.3726	588.4063	669.3566
V	880.4523	862.4417	782.4754	863.4258	863.4496	589.3304	571.3198	491.3535	572.3039
E	1009.495	991.4843	911.518	992.4684	992.4922	490.262	472.2514	392.2851	473.2354
S	1096.527	1078.516	998.55	1079.5	1079.524	361.2194	343.2088	263.2425	344.1928
V	1195.595	1177.585	1097.618	1178.569	1178.593	274.1874	256.1768	176.2105	257.1608
R	1351.697	1333.686	1253.72	1334.67	1334.694	175.119	157.1084	77.1421	158.0924

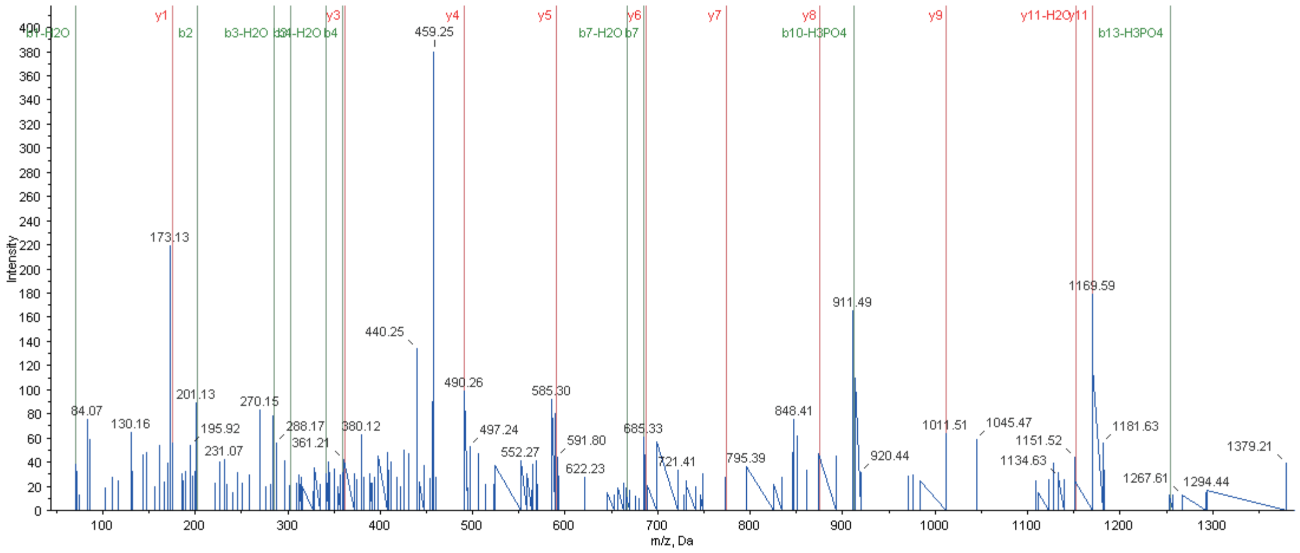

Cytoplasmic dynein

MS/MS Fragmentation of AISKDHLYGTLDPNTR found in cytoplasmic dynein 1 heavy chain 1

Fragmentation Evidence

Residue	b	b-H2O	b-H3PO4	b-NH3	b-OH	y	y-H2O	y-H3PO4	y-NH3
A	72.0444	54.0338		55.0178	55.0417	1800.924	1782.913	1702.947	1783.897
I	185.1285	167.1179	87.1516	168.1019	168.1257	1729.887	1711.876	1631.91	1712.86
S	272.1605	254.1499	174.1836	255.1339	255.1577	1616.803	1598.792	1518.826	1599.776
K	400.2554	382.2449	302.2786	383.2289	383.2527	1529.771	1511.76	1431.794	1512.744
D	515.2824	497.2718	417.3055	498.2558	498.2796	1401.676	1383.665	1303.699	1384.649
H	652.3413	634.3307	554.3644	635.3148	635.3386	1286.649	1268.638	1188.672	1269.622
L	765.4254	747.4148	667.4485	748.3988	748.4226	1149.59	1131.579	1051.613	1132.563
Y	928.4887	910.4781	830.5118	911.4621	911.486	1036.506	1018.495	938.5289	1019.479
G	985.5102	967.4996	887.5333	968.4836	968.5074	873.4425	855.4319	775.4656	856.4159
T	1086.558	1068.547	988.5809	1069.531	1069.555	816.421	798.4104	718.4441	799.3945
L	1199.642	1181.631	1101.665	1182.615	1182.639	715.3733	697.3628	617.3964	698.3468
D	1314.669	1296.658	1216.692	1297.642	1297.666	602.2893	584.2787	504.3124	585.2627
P	1411.722	1393.711	1313.745	1394.695	1394.719	487.2623	469.2518	389.2854	470.2358
N	1525.765	1507.754	1427.788	1508.738	1508.762	390.2096	372.199	292.2327	373.183
T	1626.812	1608.802	1528.835	1609.786	1609.81	276.1666	258.1561	178.1897	259.1401
R	1782.913	1764.903	1684.936	1765.887	1765.911	175.119	157.1084	77.1421	158.0924

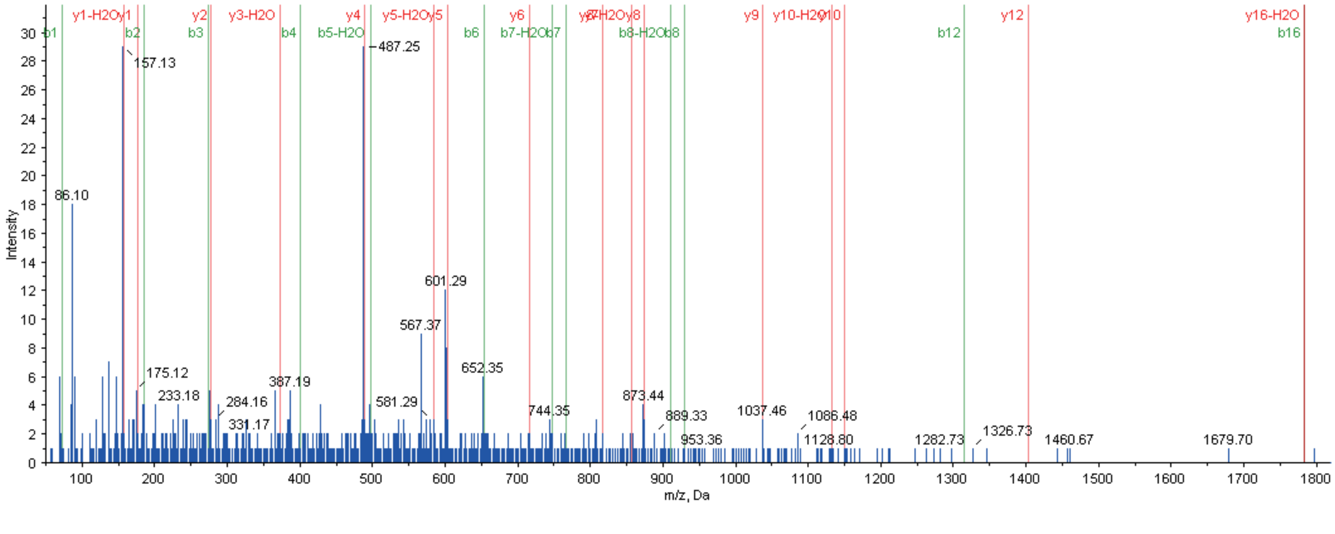

Cytoplasmic dynein 1 light intermediate chain 2

MS/MS Fragmentation of KTGSPGSPSAGGVQSTAK found in cytoplasmic dynein 1 light intermediate chain 2

Fragmentation Evidence

Residue	b	b-H2O	b-H3PO4	b-NH3	b-OH	y	y-H2O	y-H3PO4	y-NH3
K	129.1022	111.0917	31.1253	112.0757	112.0995	1616.824	1598.813	1518.847	1599.797
T	230.1499	212.1394	132.173	213.1234	213.1472	1488.729	1470.718	1390.752	1471.702
G	287.1714	269.1608	189.1945	270.1448	270.1686	1387.681	1369.671	1289.704	1370.655
S	374.2034	356.1928	276.2265	357.1769	357.2007	1330.66	1312.649	1232.683	1313.633
P	471.2562	453.2456	373.2793	454.2296	454.2534	1243.628	1225.617	1145.651	1226.601
G	528.2776	510.2671	430.3007	511.2511	511.2749	1146.575	1128.564	1048.598	1129.548
S	615.3097	597.2991	517.3328	598.2831	598.3069	1089.554	1071.543	991.5766	1072.527
P	712.3624	694.3519	614.3855	695.3359	695.3597	1002.521	984.5109	904.5446	985.4949
S	799.3945	781.3839	701.4176	782.3679	782.3917	905.4687	887.4581	807.4918	888.4421
A	870.4316	852.421	772.4547	853.405	853.4288	818.4367	800.4261	720.4598	801.4101
G	927.453	909.4425	829.4761	910.4265	910.4503	747.3995	729.389	649.4226	730.373
G	984.4745	966.4639	886.4976	967.448	967.4718	690.3781	672.3675	592.4012	673.3515
V	1083.543	1065.532	985.566	1066.516	1066.54	633.3566	615.3461	535.3797	616.3301
Q	1211.602	1193.591	1113.625	1194.575	1194.599	534.2882	516.2776	436.3113	517.2617
S	1298.634	1280.623	1200.657	1281.607	1281.631	406.2296	388.2191	308.2527	389.2031
T	1399.681	1381.671	1301.704	1382.655	1382.679	319.1976	301.187	221.2207	302.171
A	1470.718	1452.708	1372.741	1453.692	1453.716	218.1499	200.1394	120.173	201.1234
K	1598.813	1580.803	1500.836	1581.787	1581.811	147.1128	129.1022	49.1359	130.0863

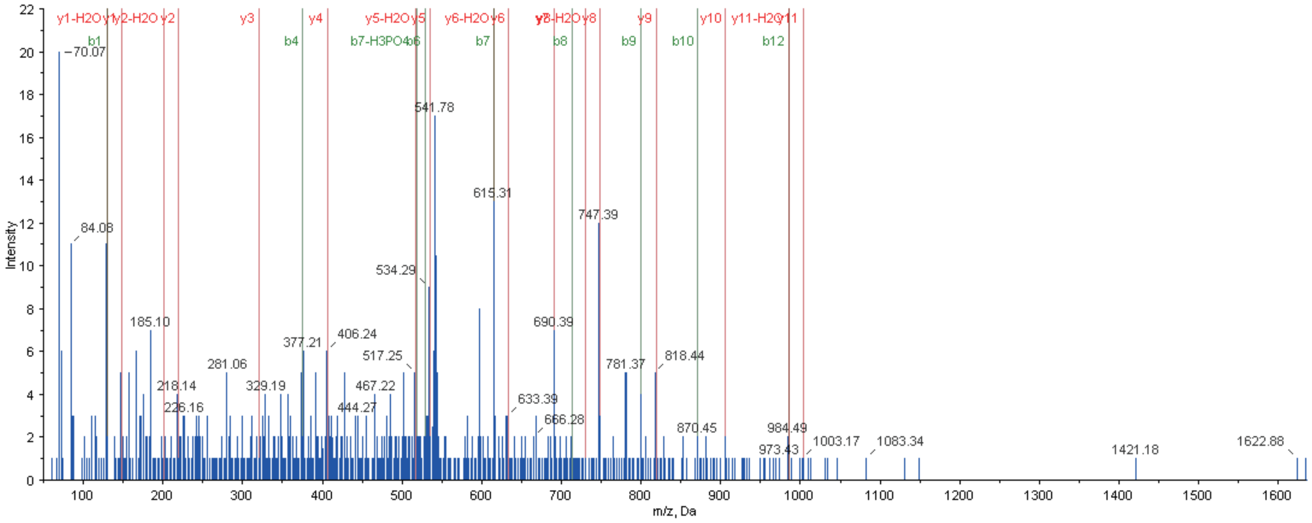

NuMA1

MS/MS Fragmentation of HELAEATASQHR found in nuclear mitotic apparatus protein 1

Fragmentation Evidence

Residue	b	b-H2O	b-H3PO4	b-NH3	b-OH	y	y-H2O	y-H3PO4	y-NH3
H	138.0662	120.0556	40.0893	121.0396	121.0634	1349.656	1331.645	1251.679	1332.629
E	267.1088	249.0982	169.1319	250.0822	250.106	1212.597	1194.586	1114.62	1195.57
L	380.1928	362.1823	282.216	363.1663	363.1901	1083.554	1065.544	985.5773	1066.528
A	451.23	433.2194	353.2531	434.2034	434.2272	970.4701	952.4595	872.4932	953.4435
E	580.2726	562.262	482.2957	563.246	563.2698	899.433	881.4224	801.4561	882.4064
A	651.3097	633.2991	553.3328	634.2831	634.3069	770.3904	752.3798	672.4135	753.3638
T	752.3573	734.3468	654.3804	735.3308	735.3546	699.3533	681.3427	601.3764	682.3267
A	823.3945	805.3839	725.4176	806.3679	806.3917	598.3056	580.295	500.3287	581.279
S	910.4265	892.4159	812.4496	893.3999	893.4237	527.2685	509.2579	429.2916	510.2419
Q	1038.485	1020.475	940.5082	1021.459	1021.482	440.2364	422.2259	342.2595	423.2099
H	1175.544	1157.533	1077.567	1158.517	1158.541	312.1779	294.1673	214.201	295.1513
R	1331.645	1313.635	1233.668	1314.619	1314.642	175.119	157.1084	77.1421	158.0924

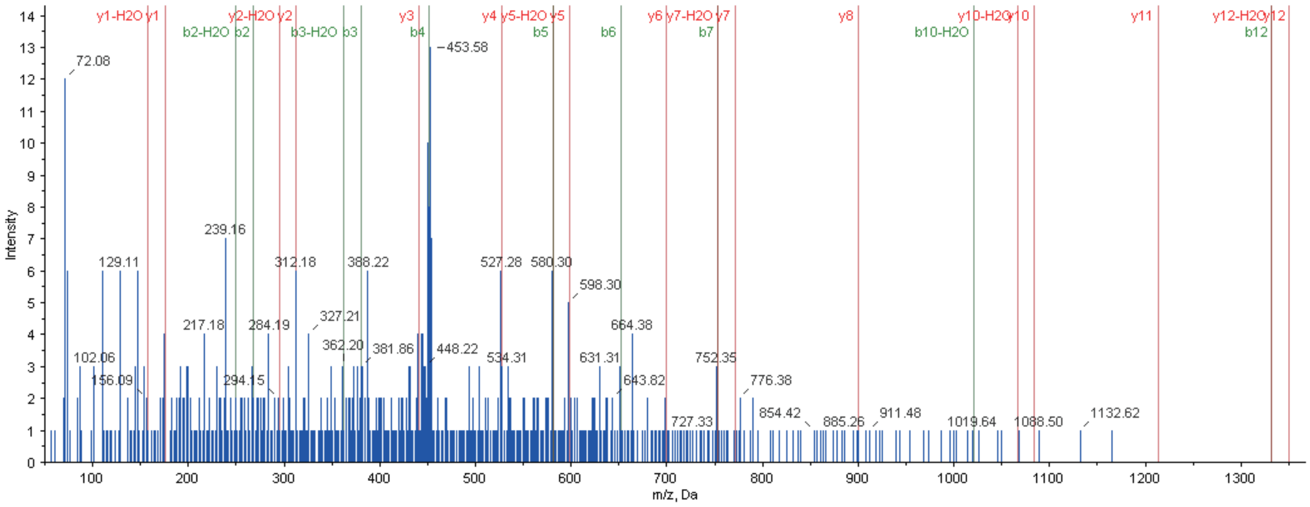

Beta-tubulin

MS/MS Fragmentation of AILVDLEPGTMDSVR found in tubulin beta-2B chain

Fragmentation Evidence

Residue	b	b-H2O	b-H3PO4	b-NH3	b-OH	y	y-H2O	y-H3PO4	y-NH3
A	72.0444	54.0338		55.0178	55.0417	1631.831	1613.82	1533.854	1614.804
I	185.1285	167.1179	87.1516	168.1019	168.1257	1560.794	1542.783	1462.817	1543.767
L	298.2125	280.202	200.2356	281.186	281.2098	1447.71	1429.699	1349.733	1430.683
V	397.2809	379.2704	299.304	380.2544	380.2782	1334.626	1316.615	1236.649	1317.599
D	512.3079	494.2973	414.331	495.2813	495.3051	1235.557	1217.547	1137.58	1218.531
L	625.3919	607.3814	527.415	608.3654	608.3892	1120.53	1102.52	1022.553	1103.504
E	754.4345	736.424	656.4576	737.408	737.4318	1007.446	989.4357	909.4693	990.4197
P	851.4873	833.4767	753.5104	834.4607	834.4846	878.4036	860.3931	780.4267	861.3771
G	908.5088	890.4982	810.5319	891.4822	891.506	781.3509	763.3403	683.374	764.3243
T	1009.556	991.5459	911.5795	992.5299	992.5537	724.3294	706.3189	626.3525	707.3029
M[Oxi]	1156.592	1138.581	1058.615	1139.565	1139.589	623.2817	605.2712	525.3048	606.2552
D	1271.619	1253.608	1173.642	1254.592	1254.616	476.2463	458.2358	378.2694	459.2198
S	1358.651	1340.64	1260.674	1341.624	1341.648	361.2194	343.2088	263.2425	344.1928
V	1457.719	1439.709	1359.742	1440.693	1440.717	274.1874	256.1768	176.2105	257.1608
R	1613.82	1595.81	1515.843	1596.794	1596.818	175.119	157.1084	77.1421	158.0924

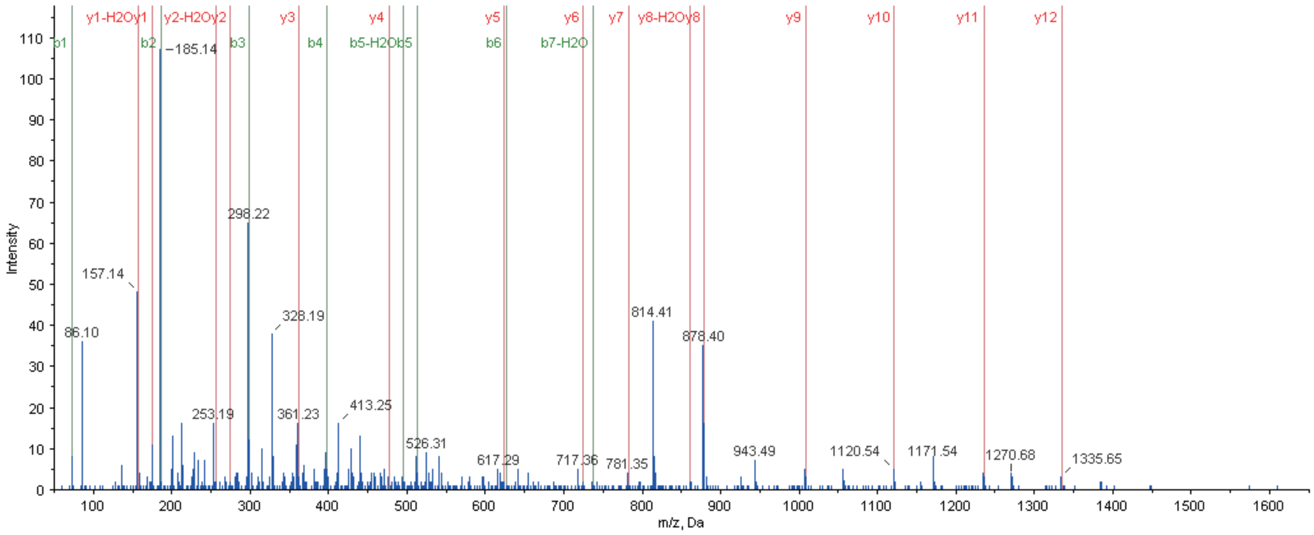

Alpha-tubulin

MS/MS Fragmentation of AFVHWYVGEGMEEGEFSEAR found in tubulin alpha-1A chain

Fragmentation Evidence

Residue	b	b-H2O	b-H3PO4	b-NH3	b-OH	y	y-H2O	y-H3PO4	y-NH3
A	72.0444	54.0338		55.0178	55.0417	2346.013	2328.003	2248.036	2328.987
F	219.1128	201.1022	121.1359	202.0863	202.1101	2274.976	2256.966	2176.999	2257.95
V	318.1812	300.1707	220.2043	301.1547	301.1785	2127.908	2109.897	2029.931	2110.881
H	455.2401	437.2296	357.2632	438.2136	438.2374	2028.839	2010.829	1930.862	2011.813
W	641.3194	623.3089	543.3425	624.2929	624.3167	1891.78	1873.77	1793.803	1874.754
Y	804.3828	786.3722	706.4059	787.3562	787.38	1705.701	1687.69	1607.724	1688.675
V	903.4512	885.4406	805.4743	886.4246	886.4484	1542.638	1524.627	1444.661	1525.611
G	960.4726	942.4621	862.4958	943.4461	943.4699	1443.569	1425.559	1345.592	1426.543
E	1089.515	1071.505	991.5383	1072.489	1072.513	1386.548	1368.537	1288.571	1369.521
G	1146.537	1128.526	1048.56	1129.51	1129.534	1257.505	1239.495	1159.528	1240.479
M[Oxi]	1293.572	1275.562	1195.595	1276.546	1276.569	1200.484	1182.473	1102.507	1183.457
E	1422.615	1404.604	1324.638	1405.588	1405.612	1053.448	1035.438	955.4714	1036.422
E	1551.657	1533.647	1453.68	1534.631	1534.655	924.4058	906.3952	826.4289	907.3792
G	1608.679	1590.668	1510.702	1591.652	1591.676	795.3632	777.3526	697.3863	778.3366
E	1737.721	1719.711	1639.745	1720.695	1720.719	738.3417	720.3311	640.3648	721.3151
F	1884.79	1866.779	1786.813	1867.763	1867.787	609.2991	591.2885	511.3222	592.2726
S	1971.822	1953.811	1873.845	1954.795	1954.819	462.2307	444.2201	364.2538	445.2041
E	2100.864	2082.854	2002.888	2083.838	2083.862	375.1987	357.1881	277.2218	358.1721
A	2171.902	2153.891	2073.925	2154.875	2154.899	246.1561	228.1455	148.1792	229.1295
R	2328.003	2309.992	2230.026	2310.976	2311	175.119	157.1084	77.1421	158.0924

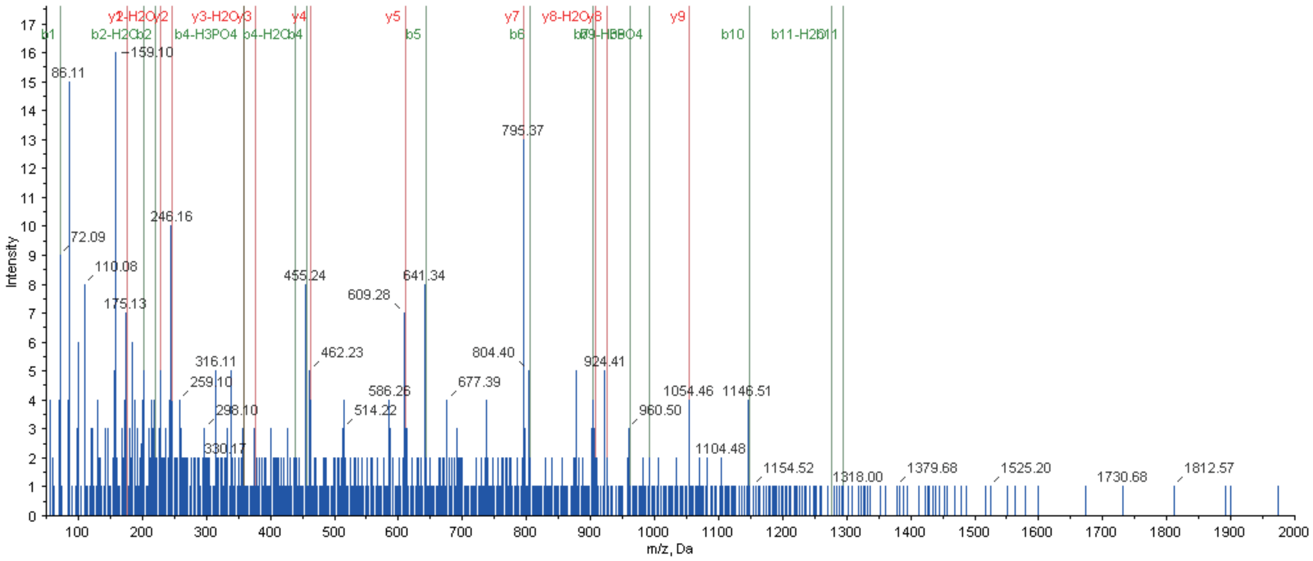

Beta III-tubulin

MS/MS Fragmentation of FWEVISDEHGIDPSGNYVGDSDLQLER found in tubulin beta-3 chain

Fragmentation Evidence

Residue	b	b-H2O	b-H3PO4	b-NH3	b-OH	y	y-H2O	y-H3PO4	y-NH3
F	148.0757	130.0651	50.0988	131.0491	131.073	3077.412	3059.402	2979.435	3060.386
W	334.155	316.1444	236.1781	317.1285	317.1523	2930.344	2912.333	2832.367	2913.317
E	463.1976	445.187	365.2207	446.171	446.1949	2744.265	2726.254	2646.288	2727.238
V	562.266	544.2554	464.2891	545.2395	545.2633	2615.222	2597.211	2517.245	2598.195
I	675.3501	657.3395	577.3732	658.3235	658.3473	2516.154	2498.143	2418.177	2499.127
S	762.3821	744.3715	664.4052	745.3556	745.3794	2403.07	2385.059	2305.093	2386.043
D	877.409	859.3985	779.4322	860.3825	860.4063	2316.038	2298.027	2218.061	2299.011
E	1006.452	988.4411	908.4747	989.4251	989.4489	2201.011	2183	2103.034	2183.984
H	1143.511	1125.5	1045.534	1126.484	1126.508	2071.968	2053.957	1973.991	2054.941
G	1200.532	1182.521	1102.555	1183.506	1183.529	1934.909	1916.899	1836.932	1917.883
I	1313.616	1295.606	1215.639	1296.59	1296.613	1877.888	1859.877	1779.911	1860.861
D	1428.643	1410.633	1330.666	1411.617	1411.64	1764.804	1746.793	1666.827	1747.777
P	1525.696	1507.685	1427.719	1508.669	1508.693	1649.777	1631.766	1551.8	1632.75
S	1612.728	1594.717	1514.751	1595.701	1595.725	1552.724	1534.713	1454.747	1535.697
G	1669.749	1651.739	1571.772	1652.723	1652.747	1465.692	1447.681	1367.715	1448.665
N	1783.792	1765.782	1685.815	1766.766	1766.79	1408.67	1390.66	1310.693	1391.644
Y	1946.856	1928.845	1848.879	1929.829	1929.853	1294.627	1276.617	1196.651	1277.601
V	2045.924	2027.913	1947.947	2028.897	2028.921	1131.564	1113.554	1033.587	1114.538
G	2102.945	2084.935	2004.969	2085.919	2085.943	1032.496	1014.485	934.5187	1015.469
D	2217.972	2199.962	2119.996	2200.946	2200.97	975.4742	957.4636	877.4973	958.4476
S	2305.004	2286.994	2207.028	2287.978	2288.002	860.4472	842.4367	762.4703	843.4207
D	2420.031	2402.021	2322.054	2403.005	2403.029	773.4152	755.4046	675.4383	756.3886
L	2533.115	2515.105	2435.139	2516.089	2516.113	658.3883	640.3777	560.4114	641.3617
Q	2661.174	2643.163	2563.197	2644.147	2644.171	545.3042	527.2936	447.3273	528.2776
L	2774.258	2756.248	2676.281	2757.232	2757.255	417.2456	399.235	319.2687	400.2191
E	2903.301	2885.29	2805.324	2886.274	2886.298	304.1615	286.151	206.1847	287.135
R	3059.402	3041.391	2961.425	3042.375	3042.399	175.119	157.1084	77.1421	158.0924

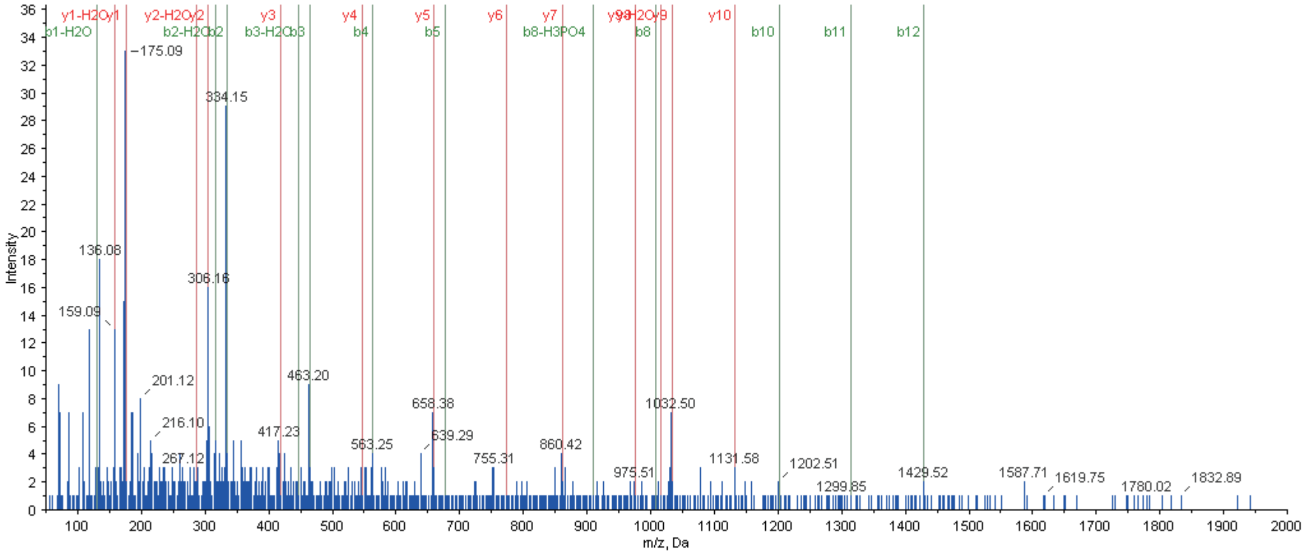

Supplement: Supplementary Figures [file srep39902-s16.pdf]
